# Smart ROS-regulating biomimetic hydrogel promotes scarless diabetic wound healing via macrophage reprogramming

**DOI:** 10.1016/j.mtbio.2026.103061

**Published:** 2026-03-27

**Authors:** Pan Du, Jin Li, Jun Pu, Shuqian Dou, Gaofei Zhang, Kongjia Wu, Shihao Deng, Qiulei Wang, Wenjun Liu

**Affiliations:** a The Second Affiliated Hospital of Kunming Medical University, Kunming, Yunnan, 650000, China; b Kunming Medical University, Kunming, Yunnan, 650500, China; c Shenzhen Smoore Technology Limited, Shenzhen, Guangdong, 518126, China

**Keywords:** Smart hydrogel, Stem cell-derived nanovesicles, Reactive oxygen species regulation, Immunomodulation, Scar-free healing

## Abstract

The dynamic management of diabetic wound healing remains a major challenge due to the vastly different therapeutic requirements across healing phases and the impaired drug delivery within the pathological microenvironment. To address this, we develop a core–shell structured microfiber composite hydrogels (Bil), inspired by the three-dimensional steel-concrete composite structures in civil engineering, that enables programmed treatment by dynamically regulating ROS and the immune microenvironment. Initially, Bil generates antibacterial ROS under laser irradiation. As the ROS-responsive shell degrades, stem cell-derived exosomes (Exos) are released to promote regeneration, while the exposed nanofibrous core scavenges ROS and facilitates the inflammation-to-proliferation transition. Furthermore, released verteporfin inhibits scar formation by blocking Engrailed-1 (En1) activation in fibroblasts. This platform provides spatiotemporal stage-adaptive therapy, significantly enhancing healing through dynamic ROS modulation, precise immune regulation, and improved Exos delivery, ultimately promoting scarless wound regeneration.

## Graphical abstract

**Figure:**
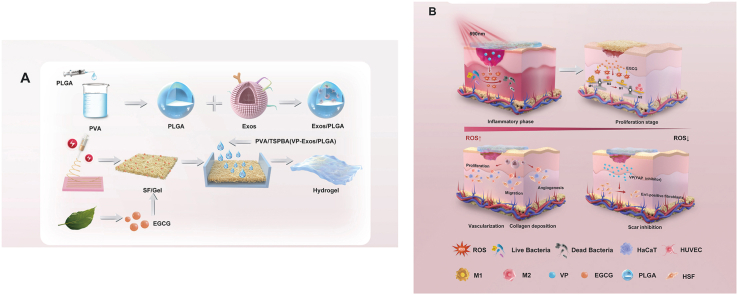

## 1 Introduction

Chronic wound management remains a formidable clinical challenge, with diabetic ulcers alone affecting over 25% of patients with diabetes mellitus worldwide [1]. The physiological wound healing cascade—hemostasis, inflammation, proliferation, and remodeling—requires precise spatiotemporal coordination between cellular responses and biochemical signaling [2]. However, pathological microenvironments in chronic wounds disrupt this orchestrated process through persistent oxidative stress and immune dysregulation [3,4]. In diabetic wounds, hyperglycemia-induced reactive oxygen species (ROS) overproduction creates a self-perpetuating vicious cycle: impaired macrophage polarization (M1-to-M2 transition), neutrophil extracellular trap (NET) overactivation, and pro-inflammatory cytokine accumulation collectively trap the healing process in chronic inflammation [5,6]. This pathophysiological stagnation leads to insufficient angiogenesis, aberrant extracellular matrix (ECM) deposition, and ultimately, dysfunctional tissue repair with fibrotic scarring [7,8]. Conventional therapeutic strategies focusing on single-phase intervention (e.g., ROS scavenging or growth factor delivery) fail to address the dynamic nature of wound microenvironments [9,10], Collectively, these limitations underscore the urgent need for intelligent systems capable of phase-adaptive regulation.

The dichotomous role of reactive oxygen species (ROS) in wound pathophysiology presents a critical therapeutic paradox, demanding sophisticated redox equilibrium management throughout healing cascades. During the inflammatory phase, controlled ROS generation (<100 μM) executes essential bactericidal functions through peroxidase-mediated pathogen lysis while activating hypoxia-inducible factor-1α (HIF-1α) to initiate angiogenesis [11,12]. Paradoxically, sustained supraphysiological ROS levels (>250 μM) induce lipid peroxidation chain reactions, triggering fibroblast senescence via p38 MAPK-γH2AX pathways and perpetuating inflammatory loops through NLRP3 inflammasome activation [13,14]. Current monodirectional interventions—including antioxidant flooding (e.g., NAC, vitamin E) or pro-oxidant therapies (e.g., H_2_O_2_-releasing scaffolds)—fail to address this temporal duality, often disrupting phase-specific redox signaling [15,16]. For instance, global ROS scavenging during re-epithelialization impairs neutrophil extracellular trap (NET) formation and VEGF-A/SDF-1α crosstalk [17,18], while excessive ROS persistence during remodeling upregulates MMP-9/COL1A1 imbalance, causing collagen fibril disorganization [19]. To resolve this therapeutic dilemma, this necessitates intelligent systems capable of phase-adaptive ROS flux control: transient ROS elevation for microbial clearance and angiogenic priming, followed by sustained antioxidant delivery to resolve inflammation and stabilize ECM architecture.

The multifaceted pathophysiology of wound healing necessitates a therapeutic strategy that transcends conventional antimicrobial approaches, demanding precise spatiotemporal regulation of reactive oxygen species (ROS) dynamics in conjunction with targeted immunomodulation. Beyond these fundamental requirements, successful tissue regeneration necessitates orchestrated activation of phase-specific bioactive components, particularly neovascularization and extracellular matrix (ECM) remodeling [20,21]. Notably, emerging evidence highlights umbilical cord-derived mesenchymal stem cell exosomes (hUC-MSCs-Exos) as pivotal mediators of regenerative processes [22]. Compared to stem cell therapies, hUC-MSCs-Exos circumvent tumorigenicity risks while delivering a complex cocktail of regenerative factors (miRNAs, cytokines, and ECM proteins) [23]. Mechanistically, hUC-MSCs-Exos enhance neutrophil phagocytosis, promote M2 macrophage polarization via TSG-6 signaling, and activate fibroblast proliferation through ERK1/2 phosphorylation [24,25]. Previous work further demonstrated their capacity to stabilize hypoxia-inducible factor 1α (HIF-1α) in keratinocytes, accelerating re-epithelialization [26]. Nevertheless, rapid enzymatic degradation and nonspecific distribution limit their therapeutic efficacy in vivo [27]. Although biomaterial carriers (e.g., hydrogels, microspheres) can prolong exosome retention, most existing systems lack the dynamic responsiveness required for phased wound healing regulation [28,29].

Inspired by the three-dimensional steel-concrete composite structures in civil engineering, we engineered a biomimetic core-shell system (Bil) through hierarchical integration of the nanofiber-reinforced "steel" core and ROS-responsive "concrete" shell (Fig. 1a). This hierarchical architecture comprises two functionally integrated compartments: (1) A reactive oxygen species (ROS)-responsive "concrete" matrix constructed through TSPBA-crosslinked polyvinyl alcohol, engineered to co-encapsulate hUC-MSCs-Exos and verteporfin (VP, YAP/TAZ signaling inhibitor). This compartment provides photodynamic bactericidal capability under laser irradiation while enabling controlled exosome release aligned with proliferative phase requirements. (2) A mechanically reinforced "steel" core fabricated via electrospinning sericin-gelatin composite fibers, strategically loaded with epigallocatechin gallate (EGCG) to establish late-phase antioxidant reservoirs for inflammatory resolution [30]. In preclinical diabetic wound models, Bil exhibited triphasic therapeutic precision through materials-guided microenvironmental reprogramming: The initial phase leveraged verteporfin-mediated ROS generation to eliminate pathogenic biofilms, followed by EGCG-driven oxidative stress attenuation and macrophage phenotype switching toward regenerative M2 polarization. Concurrently, sustained hUC-MSCs-Exos delivery enhanced the biological functions of fibroblasts and vascular endothelial cells while suppressing fibroblast-to-myofibroblast transition via YAP inhibition(Fig. 1b). This spatiotemporally programmed therapeutic modality successfully reconciled the pathognomonic inflammation-proliferation paradox in chronic wounds, as evidenced by accelerated re-epithelialization, neovascularization, and collagen matrix reorganization with reduced fibrotic scarring. The bioinspired design paradigm establishes a transformative approach for intelligent wound management, where structural mechanics-guided material engineering synchronizes antimicrobial defense, immunomodulation, and ECM remodeling across healing phases.

**Fig. 1:**
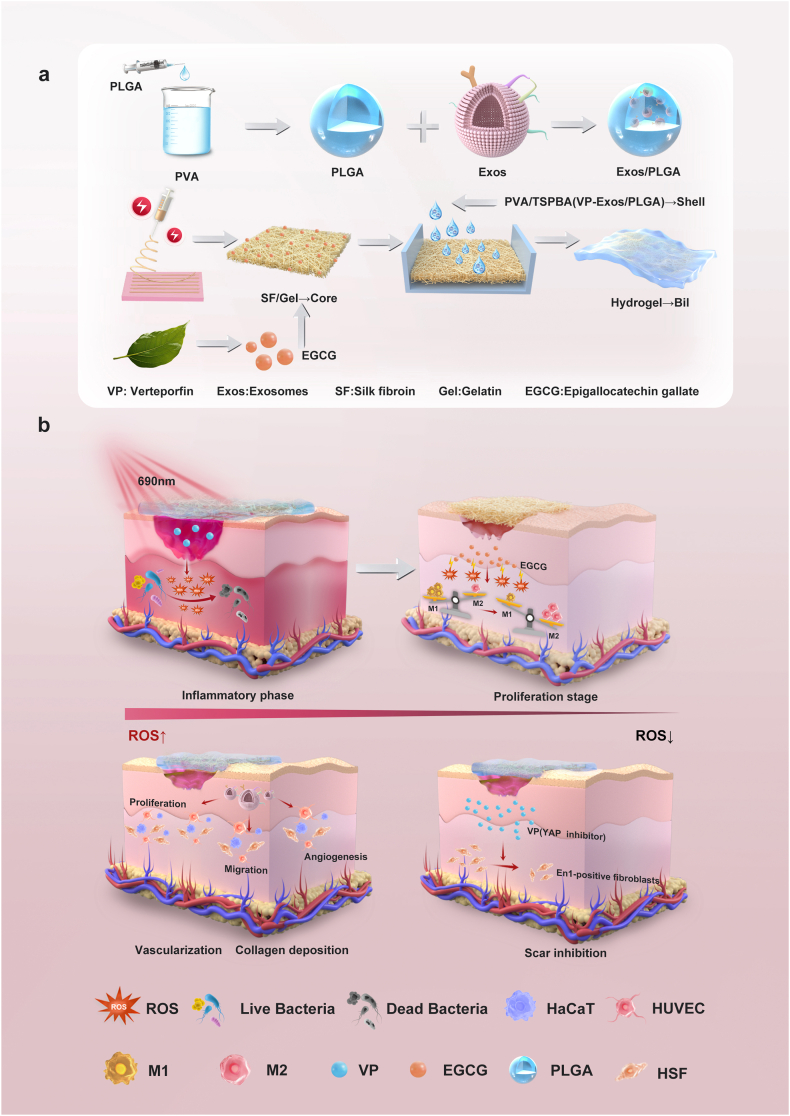
**Diagram showing the fabrication of the microfiber-composite hydrogel platform and its mechanism of action in wound healing.** (a) Schematic of the Bil composite hydrogel, consisting of an inner SF-GT electrospun layer coated with an outer PVA-TSPBA layer. (b) By orchestrating dynamic ROS regulation (scavenging and modulation), antibacterial action, and immunomodulation, the Bil hydrogel inhibits the fibroblast-associated YAP pathway, ultimately driving cell migration, angiogenesis, and scar-free wound repair.

## 2 Results and discussion

### 2.1 Preparation and characterization of bil

**Fig. 2:**
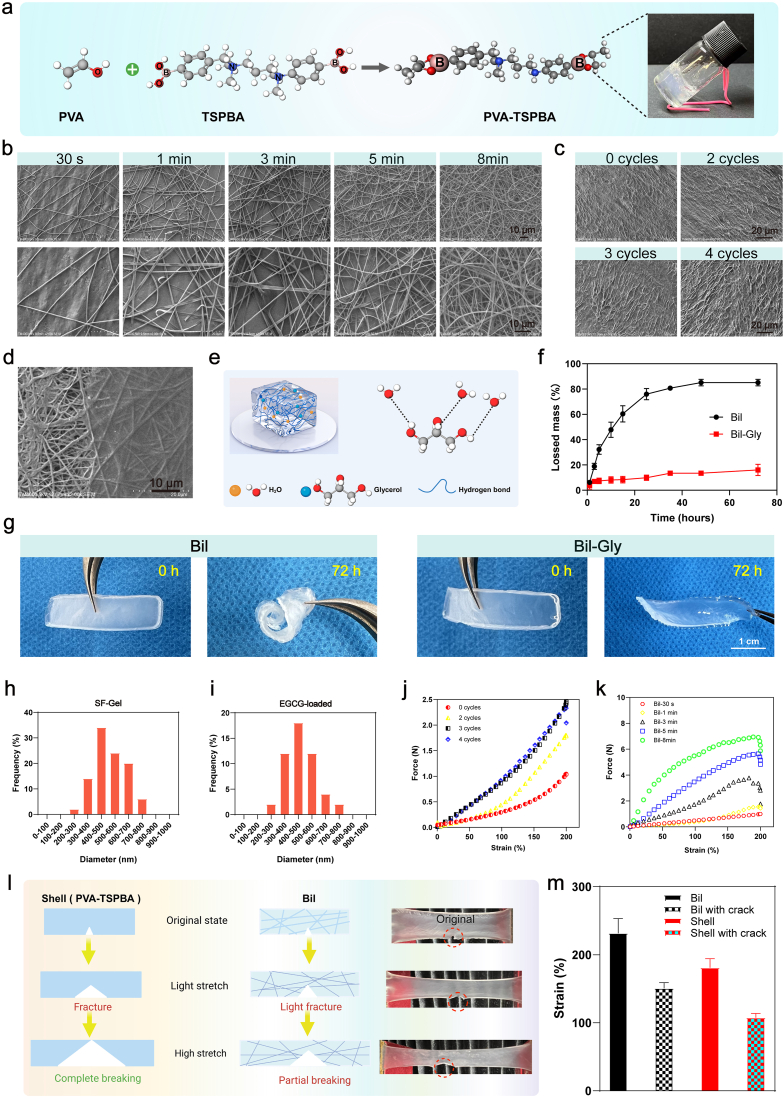
**Characterization and mechanical properties of the Bil composite hydrogel.** (a) Gelation Mechanism of PVA-TSPBA Hydrogel. (b) SEM micrographs of SF-GT nanofibers produced with different electrospinning durations (30 s, 1, 3, 5, and 8 min). (c) SEM micrographs of PVA-TSPBA hydrogels subjected to different freeze-thaw cycles. (d) SEM micrograph showing the PVA-TSPBA coating on the surface of SF-GT nanofibers. (e) Schematic illustration of the water-retention mechanism of glycerol. (f) Mass change of Bil and Bil-Gly hydrogels over time (n = 3 independent samples). (g) Macroscopic appearance of Bil and Bil-Gly after 72 h of incubation. (h, i) Statistical analysis of the diameter distribution of pristine SF-GT nanofibers and EGCG-loaded SF-GT nanofibers. (j) Stress-strain curves of PVA-TSPBA hydrogels prepared with different numbers of freeze-thaw cycles. (k) Stress-strain curves of Bil composite hydrogels with core layers fabricated using different electrospinning times. (l) Schematic illustration of the anti-tearing behavior of the Bil hydrogel. (m) Comparison of the maximum strain achieved by intact and notched Bil hydrogels versus pure PVA-TSPBA hydrogels (n = 3 independent samples). The results are shown as the mean ± standard deviation (SD). The symbols ∗p < 0.05, ∗∗p < 0.01, ∗∗∗p < 0.001.

To fabricate a microfiber-reinforced composite hydrogel with a biomimetic architectural structure, a degradable Bil shell was first prepared using polyvinyl alcohol (PVA, 2%) and the ROS-sensitive linker N^1^-(4-boronobenzyl)-N^3^-(4-boronophenyl)-N^1^,N^1^,N^3^,N^3^-tetramethylpropane-1,3-diaminium (TSPBA, 2%) at a volume ratio of 2:1. The shell formed as a result of the dual phenylboronic acid groups in TSPBA forming complexes with the diol moieties on PVA.The fully cross-linked PVA-TSPBA hydrogel exhibited a uniform semi-transparent appearance (Fig. 2a). As shown in Fig. S1a, the PVA–TSPBA hydrogel demonstrated excellent adhesive properties, allowing it to firmly adhere to various substrates. This provides a robust foundation for subsequent integration with the core layer. "The "steel layer" nanofibers (core) were fabricated via electrospinning, and their structural characteristics were systematically evaluated through scanning electron microscopy (SEM). As shown in (Fig. 2b), all nanofibers exhibited randomly oriented, uniform, and continuous bead-free morphology regardless of spinning duration. Notably, fiber density progressively increased with extended fabrication time, demonstrating precise control over matrix architecture. Comparative analysis revealed that EGCG incorporation had negligible impact on fiber dimensions, the average particle diameter for both the initial SF-Gel and the SF-Gel with added EGCG ranged from 200 nm to 800 nm (Fig. 2h and i). The engineered nanofibers featured a highly interconnected porous architecture, which facilitated efficient cell infiltration and dynamic molecular exchange with the wound microenvironment. Simultaneously, this permeable network enabled effective drainage of wound exudate, confirming suitability for chronic wound applications [31].

The "concrete layer" hydrogel (shell) was fabricated by crosslinking polyvinyl alcohol (PVA) with the ROS-responsive crosslinker TSPBA, yielding a dynamically adaptive PVA-TSPBA hydrogel system. Scanning electron microscopy (SEM) confirmed the formation of an interconnected porous network architecture, characteristic of effective polymer crosslinking (Fig. 2c). Surface analysis revealed no significant morphological changes in PVA-TSPBA after repeated freeze-thaw treatments. Cross-sectional SEM imaging unequivocally demonstrated complete and homogeneous shell-layer encapsulation of the core structure (Fig. 2d). The mechanical performance of PVA-TSPBA hydrogels under varying freeze-thaw cycles was systematically investigated. As shown in Fig. 2j, cyclic freezing-thawing treatments significantly enhanced mechanical properties, with optimal reinforcement achieved at 3-4 cycles—250% improvement in tensile strength. This nonlinear reinforcement pattern correlates with cryo-induced crystallization dynamics, where moderate cycling promotes polymer chain alignment and hydrogen bond densification, while excessive cycles (>5) induce structural defects through ice crystal overgrowth.

Compared to conventional bulk hydrogels, the thin structural design of Bil composite hydrogel increases its surface area exposure to ambient environments, rendering it susceptible to dehydration—a critical limitation that impedes wound healing by inducing excessive tissue desiccation. To address this challenge, we developed a glycerol-impregnated Bil system (Bil-Gly), where glycerol molecules form extensive hydrogen bonds with water networks [32,33], effectively suppressing free water evaporation and crystallization (Fig. 2e). As shown in Fig. 2f and g, Bil-Gly retained 80.2 ± 3.5% of its initial mass and maintained structural integrity after 72 h under ambient conditions (22 °C, 40% RH), while unmodified Bil underwent rapid dehydration—hardening within 48 h and retaining <20% mass after 72 h.

The electrospun core microfibers functioned as a structural skeleton, significantly enhancing the mechanical resilience of the composite hydrogel (Young's modulus increased by 3-fold vs homogeneous hydrogels). The mechanical performance of the Bil composite system was systematically investigated as a function of nanofiber density modulated by electrospinning duration. As demonstrated in Fig. 2k, the tensile strength exhibited strong electrospinning time-dependence, reaching optimal reinforcement at 8 min with a remarkable 720% enhancement (6.63N) compared to the baseline control (0.92N). This nonlinear reinforcement behavior originates from competing mechanisms: Shorter durations produced insufficient fiber integration, while prolonged electrospinning induced fiber agglomeration, compromising stress distribution efficiency. Strategic utilization of gelatin's aqueous solubility in the core facilitated seamless interfacial bonding between core and shell phases, thereby enhancing the composite hydrogel's structural stability under physiological shear stress.

The biomimetic core-shell architecture endows the Bil composite hydrogel with exceptional tear resistance, as quantitatively demonstrated in Fig. 2l and m. Bil exhibits a fracture strain of 232 ± 21% (151 ± 8% for pre-cracked samples), significantly surpassing the performance of pure PVA-TSPBA hydrogel (181 ± 13% strain, 108 ± 6% for pre-cracked counterparts). This enhanced tear resistance arises from synergistic reinforcement mechanisms: The embedded SF-Gel nanofibers create substantial stress transfer lengths due to their high matrix modulus ratio, effectively arresting crack propagation through three-dimensional energy dissipation. These results demonstrate that Bil exhibits superior mechanical performance for load-bearing biomedical applications.

These results demonstrate that our designed core-shell structured Bil system exhibits outstanding performance across multiple aspects. Crucially, this well-engineered architecture establishes a robust foundation for verifying the effectiveness of spatiotemporally controlled precision therapy delivery: The ROS-responsive shell provides potent antibacterial activity during the inflammatory phase, while progressively degrading in response to elevated ROS levels. Concurrently, the gelatin-stabilized core maintains sustained antioxidant release to resolve persistent oxidative stress during proliferation and remodeling phases. This dual-phase release profile precisely mirrors the dynamic demands of diabetic wound healing, as evidenced by phase-matched therapeutic coordination—rapid microbial clearance (Phase I) followed by prolonged anti-inflammatory and pro-regenerative action (Phase II).

### 2.2 Preparation and characterization of PLGA-Exos

**Fig. 3:**
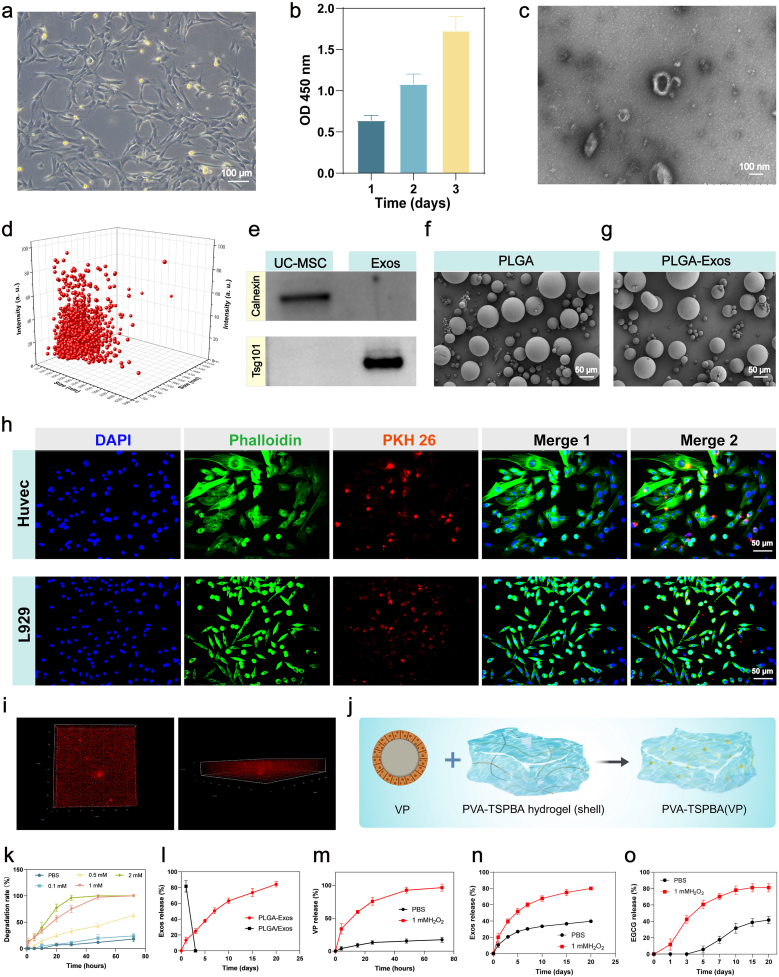
**Characterization and release of exosomes, and ROS responsiveness of the hydrogel.** (a) Morphology of human umbilical cord mesenchymal stem cells (hUC-MSCs). (b) Growth curve of hUC-MSCs over three days. (c) Transmission electron microscopy (TEM) image of hUC-MSCs-derived exosomes (hUC-MSCs-Exos). (d) Size distribution profiles of hUC-MSCs-Exos as determined by dynamic light scattering (DLS) (e) Western blot analysis of exosomal markers (e.g., CD63, TSG101). and a negative cellular marker (e.g., Calnexin) in hUC-MSCs-Exos and hUC-MSCs lysates. (f) SEM image of blank PLGA nanoparticles. (g) SEM image of hUC-MSCs-Exos-loaded PLGA nanoparticles. (h) Uptake of Exosomes by Fibroblasts and Vascular Endothelial Cells. (i) Three-Dimensional Distribution of PLGA-Encapsulated Exosomes within PVA-TSPBA Hydrogel (shell). (j) Schematic illustration of verteporfin-loaded PVA-TSPBA hydrogel. (k) Degradation behavior of Bil hydrogel in solutions with different H_2_O_2_ concentrations (n = 3 independent samples). (l) Sustained release profile of hUC-MSCs-Exos from PLGA nanoparticles over time (n = 3 independent samples). (m) Cumulative release of verteporfin (VP) from the hydrogel in PBS and H_2_O_2_ environments (n = 3 independent samples). (n) Cumulative release of hUC-MSCs-Exos from the Bil composite hydrogel in PBS and H_2_O_2_ environments (n = 3 independent samples). (0) Cumulative release of EGCG from the hydrogel in PBS and H_2_O_2_ environments (n = 3 independent samples).The results are shown as the mean ± standard deviation (SD). The symbols ∗p < 0.05, ∗∗p < 0.01, ∗∗∗p < 0.001.

Primary human umbilical cord mesenchymal stem cells (hUC-MSCs) were successfully isolated and characterized, exhibiting typical spindle-shaped fibroblastic morphology(Fig. 3a). With robust proliferative capacity as evidenced by CCK-8 assay (Fig. 3b). Subsequent exosome isolation was performed through differential ultracentrifugation of conditioned media, yielding nanoparticles that were systematically validated through multimodal characterization. Transmission electron microscopy revealed hUC-MSC-derived exosomes (hUC-MSC-Exos) exhibiting the characteristic cup-shaped morphology with intact bilayer membrane structures (Fig. 3c). Nanoparticle tracking analysis confirmed a monodisperse size distribution within the 60-160 nm range, consistent with extracellular vesicle biophysical standards (Fig. 3d). Western blot analysis further validated the exosomal biomarkers, confirming the characteristic protein profile of hUCMSCs-EVs (Fig. 3e). The vesicles showed strong positive expression of conserved exosomal markers including TSG101, while demonstrating absence of the endoplasmic reticulum contaminant protein calnexin, thus verifying the purity of our exosome isolation protocol [34]. These orthogonal analytical approaches conclusively verified the successful isolation of bona fide exosomes from hUC-MSC cultures, establishing a reliable platform for subsequent therapeutic applications. Fluorescence imaging revealed that both L929 cells and vascular endothelial cells internalized PKH26-labeled Exos after 24 h of co-culture (Fig. 3h). Furthermore, the Shell/PLGA-Exos complex, prepared using PKH26-labeled Exos, was examined under confocal microscopy, which illustrated the three-dimensional spatial distribution of Exos within the PVA-TSPBA hydrogel (shell) (Fig. 3i) (Fig. S1b).

To enhance the therapeutic efficacy of exosomes, we engineered exosome-encapsulated PLGA nanoparticles (PLGA-Exos) via a double emulsion solvent evaporation method. SEM analysis revealed that the fabricated PLGA-Exos exhibited uniform spherical morphology with smooth surfaces (Fig. 3f). Following exosome loading, the PLGA microspheres maintained their structural integrity with no significant alterations in morphology or particle size distribution (5-100 μm) (Fig. 3g). To evaluate the sustained-release capability of PLGA-Exos, we conducted a bicinchoninic acid (BCA) protein quantification assay, comparing PLGA-Exos with a physical mixture of PLGA and exosomes. The PLGA-Exos demonstrated superior sustained-release kinetics, achieving over 80% cumulative exosome release within 15 days (Fig. 3l). These results underscore the successful integration of exosomes into the PLGA matrix.

### 2.3 ROS responsiveness

During wound healing, early-stage inflammatory responses play crucial roles in pathogen clearance and host defense activation. However, excessive reactive oxygen species (ROS) exacerbate inflammation by sustaining pro-inflammatory macrophage polarization, creating a detrimental feedback loop that impairs tissue regeneration [35].To address this, we engineered Bil's immunomodulatory core-shell system with spatiotemporal control: The ROS-labile PVA-TSPBA shell undergoes programmed degradation during the early inflammatory phase (0-48 h), enabling core-derived epigallocatechin gallate (EGCG) release to scavenge ROS (Fig. 1b) and drive macrophage M2 polarization.

Systematic evaluation of the shell's ROS responsiveness revealed H_2_O_2_-dependent degradation kinetics (Fig. 3k). At 1 mM H_2_O_2_—mimicking chronic wound microenvironments—the PVA-TSPBA shell fully degraded within 40 h, synchronizing with verteporfin (VP) release kinetics (Fig. 3m). Meanwhile, To achieve sustained therapeutic delivery, PLGA-encapsulated exosomes (PLGA-Exos) were strategically integrated into the ROS-labile shell layer. This design enables the sustained release of exosomes to rely not on the ROS-responsive property of the shell layer, but rather on the controlled-release mechanism provided by the PLGA microspheres. Systematic release profiling via bicinchoninic acid (BCA) assay demonstrated microenvironment-dependent delivery kinetics. Under physiologically inert conditions (PBS, pH 7.4), exosome release plateaued at 27.1 ± 1.6% within 5 days, constrained by the non-degradable PVA-TSPBA matrix. In stark contrast, exposure to chronic wound-level ROS (1 mM H_2_O_2_) triggered programmable shell degradation, enabling PLGA microspheres to sustain exosome release over 15 days (79.9 ± 1.6% cumulative release, Fig. 3n)—a timeline precisely aligned with proliferative phase demands for angiogenesis and collagen maturation. The strategic localization of PLGA/Exos within the shell layer effectively addresses the rapid degradation-mediated exosome release and inactivation during the inflammatory phase. This engineered system achieves spatiotemporal therapeutic control across distinct healing stages while maintaining sustained exosome delivery kinetics. Concurrently, the sustained release behavior of the inner EGCG from the Bil system was investigated under two distinct conditions: PBS and 1 mM H_2_O_2_. Under physiologically inert conditions (PBS, pH 7.4), the release was restricted by the non-degradable PVA-TSPBA matrix, with EGCG only beginning to be slowly released on day 5. In stark contrast, exposure to ROS levels characteristic of chronic wounds (1 mM H_2_O_2_) triggered the programmed degradation of the shell layer, leading to the rapid release of the core-loaded EGCG by day three and a consequent antioxidant effect. (Fig. 3o).

### 2.4 Biocompatibility

The clinical viability of biomedical materials necessitates rigorous biocompatibility validation across cellular, hematological, and histological dimensions. Our systematic evaluation protocol, aligned with ISO 10993 standards, demonstrates the exceptional biosafety profile of the microfiber composite hydrogel system [36].

**Fig. 4:**
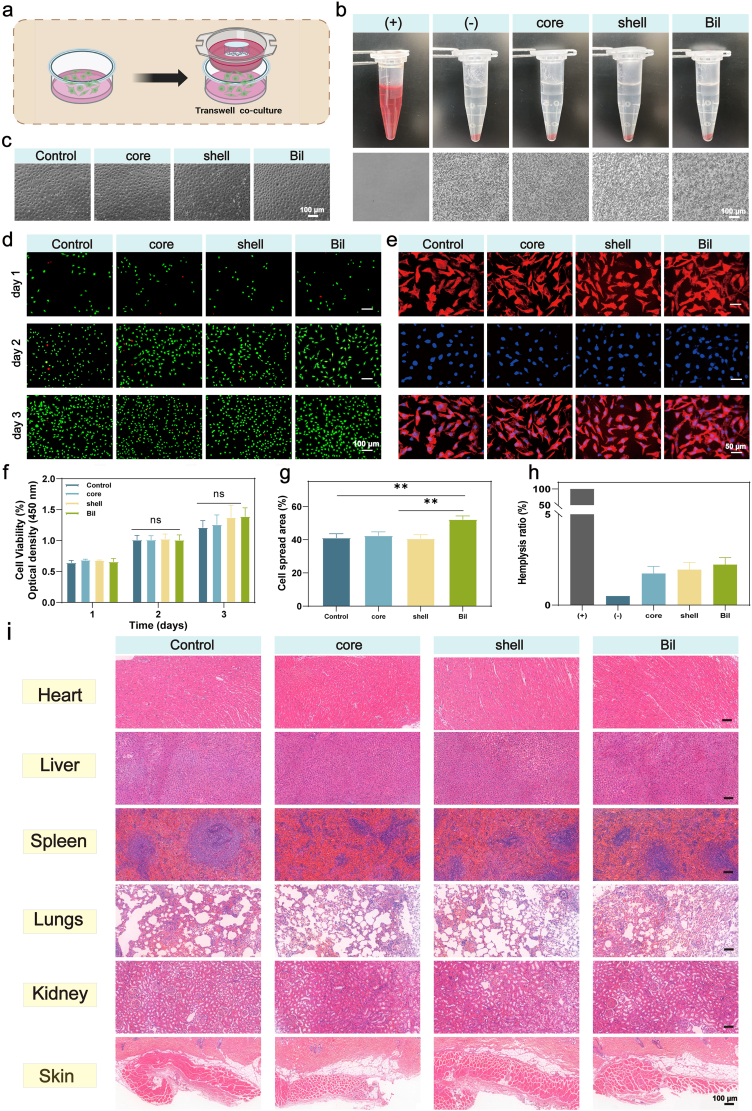
**Biocompatibility Assessment of the Hydrogel.** (a) Schematic of the Transwell co-culture system for cell–material interaction. (b) Hemocompatibility evaluation and representative images of red blood cells after contact with various samples. (c) Morphology of L929 fibroblasts after co-culture with different sample groups. (d) Live/Dead staining of cells on days 1, 2, and 3. (e) Cytoskeleton staining (Phalloidin for F-actin) illustrating cell adhesion and spreading. (f) Cell viability quantified by CCK-8 assay on days 1, 2, and 3 (n = 3 independent samples). (g) Statistical analysis of hemocompatibility results (n = 3 independent samples). (h) Quantitative analysis of cell spreading area (n = 3 independent samples). (i) Histocompatibility evaluation via H&E staining of major organs (heart, liver, spleen, lung, kidney, and skin) after treatment. The results are shown as the mean ± standard deviation (SD). The symbols ∗p < 0.05, ∗∗p < 0.01, ∗∗∗p < 0.001.

The subsequent cell compatibility experiments were conducted using the Transwell device illustrated in Fig. 4a. Given the direct blood contact requirement for wound dressings, hemolysis testing was conducted per ASTM F756-17 (human erythrocytes, 2% v/v suspension). As critical clinical thresholds: Hemolytic: >5% lysis, Non-hemolytic: <2%, Our results (Fig. 4b and g) demonstrated exceptional blood compatibility: Bil: 2.2 ± 0.4%, Core: 1.7 ± 0.4%, Shell: 2.0 ± 0.4%. These values compare favorably against commercial benchmarks: Alginate dressings: 3.5-6.2%, PU foams: 4.1-7.8% [37].Morphological examination under microscopy revealed normally shaped erythrocytes distributed across all groups (Core, Shell, and Bil). The low hemolytic activity is attributed to the material's neutral surface charge and absence of hydrophilic plasticizers, minimizing erythrocyte membrane destabilization. Following 24-h co-culture with L929 cells, all material groups exhibited normal cell morphology, as confirmed by microscopic examination (Fig. 4c). In vitro biocompatibility was quantitatively assessed using CCK-8 assays under standardized conditions (L929 fibroblasts, 1 × 10^4^ cells/cm^2^, 5% CO_2_, 37 °C). As shown in Fig. 4f, all material groups (Core, Shell, Bil) maintained excellent cell viability across 72 h, with no significant differences versus controls (DMEM-only). Notably, the incorporation of therapeutic agents (VP; EGCG) did not induce cytotoxicity, confirming dosage safety within therapeutic windows. Complementary live/dead staining at 72 h revealed <2% apoptotic cells (red fluorescence) across all groups (Fig. 4d), with confluent layers of viable cells (green fluorescence) exhibiting characteristic fibroblast morphology. Phalloidin-TRITC cytoskeletal staining (Sigma, 1:200 dilution) further confirmed physiological cellular behavior (Fig. 4e). All group fibroblasts displayed well-organized actin stress fibers and extended filopodia, indicative of favorable material-cell interactions. Quantitative analysis demonstrated enhanced cellular spreading in the Bil group compared to core andshell groups, indicating superior substrate interaction (Fig. 4h). Systemic biocompatibility was investigated through 14-day subcutaneous implantation in C57 mice. Histopathological evaluation via H&E staining of major organs (heart, liver, spleen, lung, kidney, skin) revealed no signs of inflammation, necrosis, or architectural distortion in any treatment group after 14-day implantation (Fig. 4i).

Through multidisciplinary validation—encompassing ISO 10993-5 (cytotoxicity), 10993-4 (hemocompatibility), and 10993-6 (implantation effects)—we demonstrate that the Core, Shell, and Bil systems meet Class III medical device biocompatibility requirements. Their ability to maintain tissue homeostasis while delivering therapeutic payloads positions these microfiber hydrogels as transformative candidates for chronic wound management, addressing a critical unmet need in regenerative medicine.

### 2.5 Effects on fibroblasts and vascular endothelial cells

Fibroblasts orchestrate tissue repair through coordinated migration, proliferation, and extracellular matrix (ECM) remodeling. Guided by chemotactic cues (e.g., PDGF, TGF-β), fibroblasts migrate into the wound bed via integrin-mediated adhesion and matrix metalloproteinase (MMP)-driven ECM degradation. Proliferation, activated by FGF and EGF signaling, expands the fibroblast population to rebuild damaged tissue [38,39]. Concurrently, fibroblasts synthesize and secrete collagen I/III (80–90% of total ECM), forming a provisional matrix that supports angiogenesis and keratinocyte migration. TGF-β1 further induces α-smooth muscle actin (α-SMA) expression, enabling contractile force generation for wound closure. Crucially, balanced collagen deposition (regulated by TIMPs/MMPs) and fibroblast-to-myofibroblast transition prevent pathological fibrosis [40]. Dysregulation in chronic wounds manifests as impaired migration (CXCR4↓) and excessive MMP-9 activity, highlighting fibroblasts as therapeutic targets for biomaterial-driven healing modulation [41].

**Fig. 5:**
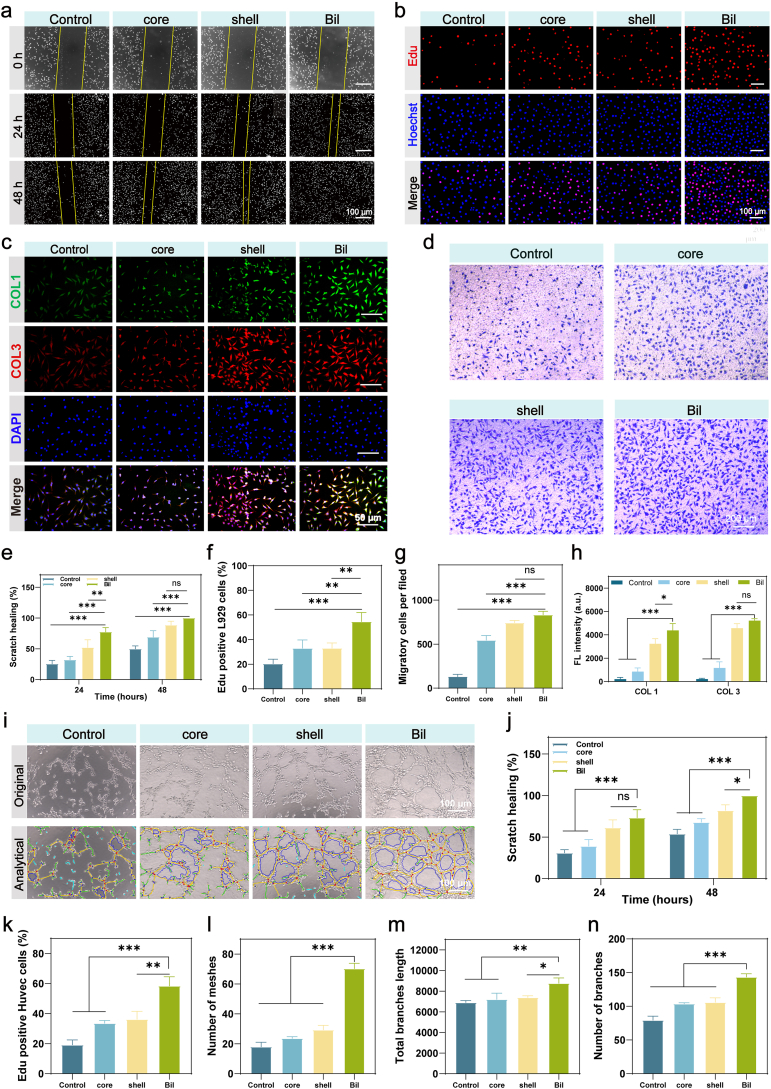
**Effects on key resident skin cells – fibroblasts and Vascular Endothelial Cells.** (a) Representative images showing fibroblast migration under different treatment conditions. (b) Evaluation of fibroblast proliferation using EdU staining across experimental groups. (c) Influence of different treatments on collagen secretion by fibroblasts. (d) Transwell assay assessing fibroblast migration in response to various samples. (e) Statistical analysis of L929 wound healing (scratch) assay (n = 3 independent samples). (f) Quantitative analysis of EdU-based fibroblast proliferation (n = 3 independent samples). (g) Statistical summary of Transwell migration assay results (n = 3 independent samples). (h) Fluorescence-based quantification of fibroblast collagen secretion in response to various treatments (n = 3 independent samples). (i) Representative images of in vitro tube formation under different treatment conditions. (j) Statistical analysis of Huvecs wound healing (scratch) assay data (n = 3 independent samples). (k) Quantitative analysis of EdU-based Huvecs proliferation (n = 3 independent samples). (l) Statistical analysis of the number of branches in tube formation assays (n = 3 independent samples). (m) Statistical analysis of the total branch length in endothelial tube networks (n = 3 independent samples). (n) Statistical analysis of the number of meshes formed in tube formation assays (n = 3 independent samples). The results are shown as the mean ± standard deviation (SD). The symbols ∗p < 0.05, ∗∗p < 0.01, ∗∗∗p < 0.001.

The regenerative capacity of the microfiber composite hydrogel was systematically evaluated through three key biological processes: fibroblast migration, proliferation, and extracellular matrix (ECM) remodeling. Quantitative analysis of cell migration using scratch wound healing assays (L929 fibroblasts, 1% FBS chemotactic gradient) revealed striking differences between groups. As shown in Fig. 5a, the Bil group demonstrated significantly enhanced wound closure efficiency, achieving 77.4 ± 7% scratch closure at 24 h—a threefold increase over control groups (25.5 ± 5.7%). The core and shell groups exhibited intermediate closure rates of 32.1 ± 5.5% and 52.2 ± 12.4%, respectively. A statistically significant difference was observed between the Bil group and the other three groups. By 48 h, complete wound closure (100%) was observed in the Bil group, while the core and shell groups reached 69 ± 10.1% and 88.6 ± 6.2%, respectively (Fig. 5e). These findings were further validated by Transwell migration assays (Fig. 5d and g), which showed that the Bil group induced significantly higher cell migration (834 ± 39 cells/field) compared to both core (545 ± 53 cells/field) and shell (743 ± 26 cells/field) groups. The Bil group exhibited statistically significant differences compared to both the Control and core groups. Both the shell and Bil groups exhibited significant promotional effects on fibroblast migration, with the Bil group displaying a limited yet discernible advantage. This enhancement in migratory capacity is attributable to exosomal components encapsulated within the shell and bilayer structures.

Cell proliferation was quantitatively analyzed using EdU staining across experimental groups, with representative results shown in Fig. 5b and f. The bilayer (Bil) microfiber composite hydrogel group demonstrated significantly higher EdU-positive rates (54.6 ± 7.4%) compared to core (33.07 ± 6.7%) and shell (33.1 ± 4.2%) groups, indicating enhanced L929 fibroblast proliferation. This proliferative enhancement correlated with the exosomal components encapsulated in both shell and bilayer constructs.

Collagen serves as the principal structural determinant of extracellular matrix (ECM) through its hierarchically organized fibrillar architecture. This three-dimensional network not only maintains dermal hydration homeostasis by stabilizing the aqueous reservoir but also provides biomechanical support for epidermal cellular constituents. Immunofluorescence analysis revealed enhanced collagen deposition in treatment groups, with bilayer (Bil)-treated L929 fibroblasts exhibiting the most pronounced type I/III collagen co-expression (Fig. 5c and h). Of note, type I collagen (COL1) and type III collagen (COL3) exhibited a high degree of co-localization in the regenerative area, which is consistent with their simultaneous synthesis and co-deposition by fibroblasts during extracellular matrix remodeling in wound healing.

Cutaneous angiogenesis plays a pivotal role in tissue regeneration by orchestrating dual biological functions: (1) facilitating nutrient/oxygen delivery to meet the metabolic demands of proliferating cells, and (2) establishing functional vascular networks that serve as structural scaffolds for re-epithelialization [42]. The density and architectural integrity of neo-vasculature directly determine healing kinetics and quality, particularly through paracrine signaling (e.g., VEGF, FGF-2) and hemodynamic regulation. Effective revascularization requires coordinated endothelial cell migration, proliferation, and lumen formation—processes critically regulated by biomaterial-mediated biochemical signaling [43,44].

Scratch wound assays with HUVECs revealed distinct migratory capacities across groups (Fig. S2a). The bilayer (Bil) hydrogel achieved complete wound closure (100%) within 48 h, representing a 1.9-fold improvement over controls (53.8 ± 5.4%). Intermediate closure rates were observed in core (67.9 ± 4.1% vs control) and shell (82.1 ± 6.7%) groups (Fig. 5j). EdU proliferation assays further confirmed Bil's bioactivity. The composite hydrogel induced 58.4 ± 6.1% EdU^+^ cells, significantly surpassing both core (33.6 ± 1.8%) and shell (36.2 ± 5.3%) groups. This mitogenic effect correlated with sustained Exos release activating ERK1/2 phosphorylation (Fig. S3a) (Fig. 5k). Tubulogenesis assays quantitatively demonstrated Bil's vascular engineering capacity (Fig. 5i). HUVECs self-organized into complex networks with 70.3 ± 3.5 branches/field (3.9 × control's 18 ± 3) (Figs. 5l) and 8769 ± 506 μm total length (1.27 × control's 6908 ± 184 μm) (Fig. 5m). Branch point analysis revealed 143 ± 5 nodal junctions in Bil groups versus 79 ± 6 in controls (Fig. 5n), indicating enhanced angiogenic complexity. Mechanistically, immunofluorescence confirmed Bil-induced VEGF overexpression (13.6-fold intensity increase vs control) through exosome-mediated Notch1 signaling activation (Fig. S4a and b).

The bilayer hydrogel demonstrates remarkable angiogenic potential through quadra-modal regulation: (1) Accelerated endothelial migration; (2) Enhanced proliferation through biochemical signaling; (3) Stabilized tubular networks; (4) VEGF upregulation via exosomal delivery. This coordinated modulation of vascularization phases—migration, proliferation, morphogenesis, and paracrine signaling—validates its efficacy in neovascularization, positioning it as a next-generation regenerative material.

### 2.6 Evaluation of ROS regulation by bil

**Fig. 6:**
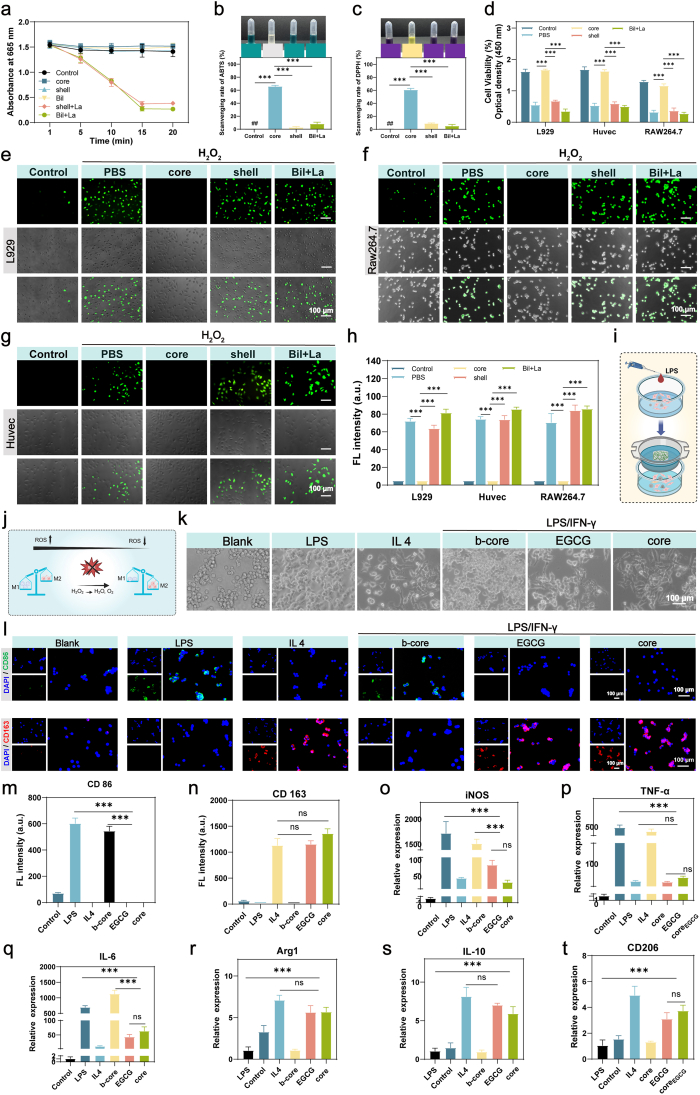
**The Dual Role of Bil Hydrogel: Dynamic ROS Regulation and Immunomodulation.** (a) Total ROS generation profiles under laser irradiation, as quantified by a methylene blue (MB) degradation assay (n = 3 independent samples). (b) Quantitative ABTS˙^+^ scavenging efficiency across sample groups. Intracellular ROS levels after laser exposure, measured using a DCFH-DA fluorescent probe. (c) Quantitative DPPH scavenging efficiency across sample groups. (d) Cell viability under H_2_O_2_-induced oxidative stress following co-culture with different sample (n = 3 independent samples). (e) Antioxidant capacity of different samples evaluated via intracellular DCFH-DA fluorescence quenching (L929). (f) Antioxidant capacity of different samples evaluated via intracellular DCFH-DA fluorescence quenching (RAW264.7). (G) Antioxidant capacity of different samples evaluated via intracellular DCFH-DA fluorescence quenching (Huvec). (h) Statistical analysis of normalized DCFH-DA fluorescence intensity in treated cells (n = 3 independent samples). (i) Experimental setup for co-culture of RAW 264.7 macrophages with the Core-layer material. (j) Schematic diagram illustrating the proposed mechanism of Core-layer-induced macrophage polarization via ROS scavenging in RAW 264.7 cells. (k) Morphology of RAW 264.7 macrophages under LPS stimulation after co-culture with the Core-layer treatment. (l) Representative immunofluorescence images of CD86^+^ (green, M1 marker) and CD163^+^ (red, M2 marker) macrophages under inflammatory conditions. (m, n) Quantitative analysis of CD86 and CD163 fluorescence intensity (n = 3 independent samples). (o-q) qPCR evaluation of pro-inflammatory gene expression (iNOS, TNF-α, IL-6) in RAW 264.7 cells treated with different samples (n = 3 independent samples). (r–t) qPCR evaluation of anti-inflammatory gene expression (ARG-1, IL-10, CD206) across treatment groups (n = 3 independent samples). The results are shown as the mean ± standard deviation (SD). The symbols ∗p < 0.05, ∗∗p < 0.01, ∗∗∗p < 0.001.

Physiological levels of reactive oxygen species (ROS, 1–10 nmol H_2_O_2_) play critical roles in wound healing by stimulating endothelial proliferation for angiogenesis and recruiting immune cells for pathogen defense [45]. However, excessive ROS (>100 nmol H_2_O_2_) induce oxidative damage to DNA, membranes, and biomolecules, impairing cellular function and delaying healing [46]. To address this contradiction and achieve biphasic ROS modulation, we first evaluated the total ROS generated under laser irradiation by monitoring the degradation of methylene blue (MB) [47]. MB exhibits a distinct absorption peak at ≈ 665 nm, and its characteristic blue color diminishes upon electron transfer in the presence of ROS, forming colorless 3,7-dimethylaminophenthiazine ions. Consequently, a reduction in absorbance at 665 nm directly correlates with increased ROS production. During prolonged incubation, the Core, Shell, and Bil groups displayed negligible changes in absorbance compared to the phosphate-buffered saline (PBS) control (denoted as Blank), indicating minimal ROS formation under irradiation. In stark contrast, the Shell + La and Bil + La groups exhibited a significant time-dependent decrease in absorbance intensity upon laser exposure, unambiguously demonstrating robust ROS generation by the Bil system under La irradiation (Fig. 6a).

The free radical scavenging potential of the composite hydrogel system was quantitatively assessed through standardized ABTS^+^and DPPH^+^assays. As shown in Fig. 6b and c, Core group demonstrated superior scavenging capacities compared to Shell architectures, achieving ABTS^+^radical inhibition rates of 65.65 ± 1.91%, (vs. 2.69 ± 1.46% for Shell and 8.08 ± 2.76% for Bil + La). Similarly, DPPH^+^assays revealed enhanced scavenging efficacy in Core (60.55 ± 2.58%), group, outperforming shell and Bil + La variants by 2.6 to 3.2-fold. This robust extracellular antioxidant activity, attributed to the ortho-dihydroxyphenyl moieties in EGCG (–OH groups at C3' and C4' positions) that donate hydrogen atoms to neutralize free radicals via hydrogen atom transfer (HAT), coupled with its electron-rich aromatic system enabling single-electron transfer (SET) to quench reactive species [48].

We measured cytoprotective effects Under Oxidative Stress by using CCK8 to test cell viability in each group. The results demonstrated the hydrogel's protective efficacy against H_2_O_2_-induced cytotoxicity (500 μM, 24 h). Core treatments significantly restored viability in HUVECs (2.19 ± 0.08%), L929 (1.98 ± 0.11%), and RAW264.7 cells (1.36 ± 0.07%) compared to H_2_O_2_-damaged controls (0.53 ± 0.08%, 0.55 ± 0.09%, 0.32 ± 0.06%, respectively) (Fig. 6d). This cytoprotection correlated with sustained EGCG release. The intracellular ROS-scavenging capacity of the microfiber composite hydrogel was further evaluated in skin-resident cells (HUVECs, L929 fibroblasts, and RAW264.7 macrophages) using the fluorescent probe DCFH-DA. Confocal microscopy revealed intense green fluorescence in L929 fibroblasts (Fig. 6e), RAW264.7 macrophages (Fig. 6f), and HUVECs (Fig. 6 g) treated with H_2_O_2_ (PBS, Shell and Bil + La groups). In contrast, the Core group exhibited a significant reduction in fluorescence intensity (Fig. 6h), thereby confirming the EGCG-mediated neutralization of reactive oxygen species (ROS) at the cellular level.

To visually examine the generation and scavenging of intracellular reactive oxygen species, we employed the fluorescent probe DCFH-DA. Experimental groups were set as follows: control, shell alone, shell + La, shell + La + b-core, and shell + La + core. This setup was designed to investigate the effect of laser irradiation of the shell layer on ROS levels in L929 cells, and to track the subsequent dynamic process of ROS scavenging upon EGCG release from the core layer.

Confocal microscopy revealed intense green fluorescence in both the shell + La and shell + La + b-core groups. In contrast, the fluorescence intensity was markedly reduced in the shell + La + core group (Fig. S5a). These findings indicate that laser irradiation of the shell layer robustly induces a state of cellular oxidative stress, which is then effectively counteracted by the EGCG released from the core.

Our results demonstrate the shell layer's pronounced ROS-generating capability synergistically integrated with the core layer's potent ROS-eliminating capacity, establishing a functionally complementary system for biphasic oxidative stress management and highlighting the system's dual functionality in dynamic ROS regulation. Combined with the previously demonstrated ROS-responsive degradation of the shell layer under oxidative conditions, this composite hydrogel enables programmable ROS modulation to align with distinct phases of wound healing: the outer shell layer generates bactericidal ROS upon laser irradiation during the early inflammatory stage, effectively combating microbial infection. Subsequently, the progressive degradation of the shell component over 48 h gradually exposes the core layer, which actively neutralizes excessive ROS to mitigate oxidative stress, thereby promoting cellular proliferation and tissue regeneration. Such spatiotemporally controlled ROS management underscores the material's potential as an advanced oxidative stress-modulating dressing for diabetic and chronic wound therapy, offering stage-adaptive therapeutic precision.

### 2.7 Modulation of immune response

Macrophages, pivotal regulators of immune responses and wound repair, exhibit remarkable plasticity between pro-inflammatory M1 (classically activated) and pro-regenerative M2 (alternatively activated) phenotypes [49]. In diabetic foot ulcers, hyperglycemia-induced oxidative stress perpetuates M1 polarization, creating a pathological feedback loop that impairs healing, as depicted in Fig. 6j [50]. Using the experimental setup illustrated in Fig. 6i, we systematically evaluated the material-mediated regulation of RAW 264.7 macrophage polarization. This study implemented a ROS-neutralizing strategy during the proliferative phase to reprogram macrophage phenotypes, thereby effectively resolving chronic inflammation through targeted immunomodulation.

Morphological analysis of RAW 264.7 macrophages revealed distinct phenotype-specific architectures. EGCG and core groups induced elongated spindle-shaped cells with extended pseudopods—a hallmark of M2 polarization—mimicking IL-4-treated positive controls. In contrast, core treatment generated amoeboid "sunflower-like" morphologies with dense pseudopods, characteristic of LPS-stimulated M1 phenotypes (Fig. 6k). The polarization dynamics of macrophages under inflammatory conditions were systematically evaluated through immunofluorescence staining. Immunofluorescent analysis of IFN-γ/LPS-stimulated macrophages revealed distinct phenotype-specific marker expression patterns: EGCG and core treatments significantly enhanced CD163^+^ (M2) signal intensity (Fig. 6l and n) while suppressing CD86^+^ (M1) expression (Fig. 6l and m) compared to controls, indicative of a robust M2-polarizing effect. Notably, while the EGCG and core constructs showed significant immunomodulatory effects, the core treatment exhibited no detectable alteration in CD86^+^ cell populations. This indicates that EGCG is the pivotal component responsible for the immunomodulatory activity within this composite system. Further transcriptomic profiling by RT-qPCR corroborated these findings at the mRNA level. Analysis of macrophage polarization state revealed that both EGCG and core constructs mediated a distinct phenotypic reprogramming, characterized by the marked downregulation of pro-inflammatory M1 markers (iNOS, TNF-α, IL-6) (Fig. 6o–q) whereas promoting the expression of anti-inflammatory M2 markers (Arg1, IL-10, CD206; Fig. 6r–t) relative to the control. This bidirectional regulation highlights core's capacity to reprogram macrophage phenotypes under diabetic wound conditions. Our experimental results demonstrate that epigallocatechin gallate (EGCG) exerts a pivotal anti-inflammatory function within the microfiber-integrated bilayer hydrogel system. Coupled with the previously validated reactive oxygen species (ROS)-responsive behavior of the shell layer, these findings conclusively reveal that the bilayer (Bil) system aligns with the chronobiology of wound healing. Specifically, the system achieves phase-specific macrophage repolarization during the proliferative stage via temporally controlled EGCG release. This spatiotemporally orchestrated modulation of macrophage polarization—targeting inflammatory-to-regenerative phenotypic switching—establishes the Bil hydrogel as a paradigm-shifting therapeutic platform. By synchronizing immunoregulatory cues with the dynamic pathophysiology of diabetic wounds, it addresses chronic inflammation and accelerates healing through mechanism-driven microenvironment reprogramming.

### 2.8 Antibacterial properties

Cutaneous bacterial infections impair healing by (1) releasing endotoxins (e.g., LPS) that directly damage tissue via caspase-3 activation, (2) inducing neutrophil extracellular trap (NET) overproduction causing collateral tissue destruction, and (3) secreting virulence factors (e.g., α-hemolysin) that suppress fibroblast collagen synthesis and keratinocyte migration. The resultant cytokine storm (IL-6, TNF-α) perpetuates inflammation while hypoxia-inducible factor (HIF-1α) degradation stalls angiogenesis. These factors lead to an inflammation-protein hydrolysis cascade that causes significant prolongation of epithelialization in infected wounds [[51], [52], [53]].

**Fig. 7:**
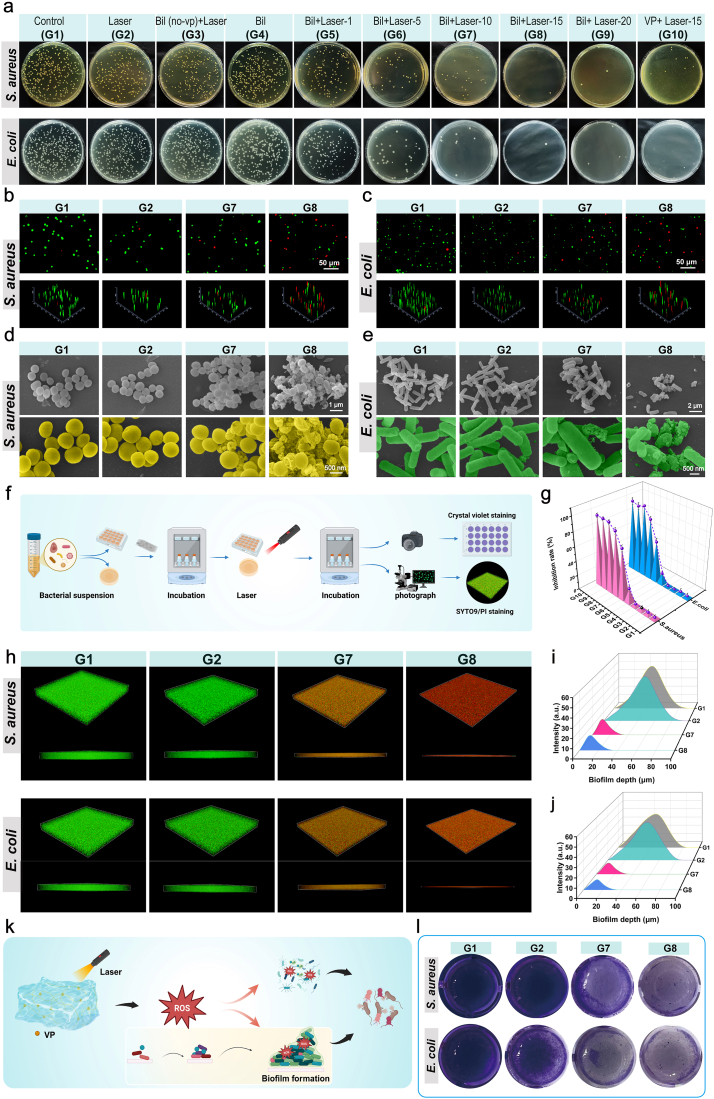
**Antibacterial Function of the Bil Composite Hydrogel.** (a) Images of *E. coli* and *S. aureu*s colonies on solid LB agar plates after treatment with different samples. (b, c) Fluorescence micrographs of live (green)/dead (red) bacteria following co-culture with various treatments. (d, e) Representative scanning electron microscopy (SEM) images of *Escherichia coli* and *Staphylococcus aureus* following various treatments. (f) Experimental flowchart for investigating the impact of composite materials on bacterial biofilms. (g) Statistical analysis of the bactericidal efficacy against *S. aureus* and *E. coli* across groups (n = 3 independent samples). (h) Live/dead staining images of bacteria in mature biofilms after treatments. (i, j) The quantified fluorescence intensity versus biofilm Depth (n = 3 independent samples). (k) Schematic illustration of the antibacterial mechanism of the Bil composite hydrogel.(l) Crystal violet staining images of bacterial biofilms after treatment with different samples. The results are shown as the mean ± standard deviation (SD). The symbols ∗p < 0.05, ∗∗p < 0.01, ∗∗∗p < 0.001.

The antimicrobial efficacy was systematically evaluated using ISO 20743-standardized plate colony counting assays against clinically prevalent strains: gram-positive *Staphylococcus aureus* (*S. aureus*) and gram-negative *Escherichia coli* (*E. coli*). Bacterial suspensions were standardized to 1 × 10^6^ CFU/mL and co-cultured with material under physiological conditions (37 °C, 5% CO_2_). As shown in Fig. 7a, In the control (PBS-treated), laser-only, Bil(no-VP) + Laser (laser-irradiated blank Bil), and Bil-only (Bil without laser irradiation) groups, substantial bacterial proliferation was consistently observed across all conditions. Simultaneously, a time-resolved evaluation of the bilayer hydrogel's photodynamic activity revealed a strong dependence on irradiation duration. Strikingly, the composite hydrogel groups subjected to extended laser irradiation (15-min [G8] and 20-min [G9] exposure) showed nearly 100% inhibition towards Gram-positive *Staphylococcus aureus* (*S. aureus*), Gram-negative *Escherichia coli* (*E. coli*), (Fig. 7g), which is attributed to cumulative reactive oxygen species (ROS) generation leading to bacterial membrane disruption. Notably, under 15-min laser irradiation, verteporfin-only demonstrated a bactericidal rate approaching complete efficacy (≈97%), confirming its role as the primary active component and elucidating the underlying mechanism responsible for the observed antibacterial effects. The above findings were further confirmed by bacterial viability staining with SYTO 9/PI (Fig. 7b and c). Ultrastructural analysis by scanning electron microscopy (SEM) revealed well-preserved microbial morphology in both the control and Laser groups, with *Staphylococcus aureus* and *Escherichia coli* exhibiting intact and smooth surfaces. In stark contrast, treatment with the Bil hydrogel followed by laser irradiation (690 nm, 15 min) induced bacterial pronounced disintegration, characterized by nanoscale pore formation, cytoplasmic leakage, and ultimate structural collapse (Fig. 7d and e).

Biofilm-embedded bacteria, encapsulated within self-secreted extracellular polymeric substances (EPS) containing polysaccharides, proteins, and eDNA, exhibit 10^3^-10^4^-fold enhanced antibiotic resistance through restricted drug penetration and persister cell formation [54]. The experimental procedure depicted in Fig. 7f was employed to investigate the effect of the composite hydrogel on bacterial biofilms. We evaluated material efficacy against 24-h immature biofilms of *S. aureus* and *E. coli* using CLSI-standardized crystal violet assays. As depicted in anti-biofilm experiments, the treatment of G8 showed higher inhibitive efficacy than G1, G2 and G7 towards both immature biofilms formed by the *S. aureus* and *E. coli* (Fig. 7l). Quantitative spectrophotometry (590 nm OD) demonstrated that 15-min laser-irradiated Bil (690 nm, 25 mW/cm^2^) achieved complete biofilm inhibition (*S. aureus*: 0.4 ± 0.27 OD vs 1.89 ± 0.32control; *E. coli:* 0.63 ± 0.25 OD vs 1.90 ± 0.24control), outperforming Laser-only (*S. aureus*: 1.89 ± 0.27 OD; *E. coli*:1.849 ± 0.18 OD) (Fig. S6a and b). Furthermore, results from bacterial biofilm live/dead staining revealed intense red fluorescence throughout the entire 3D biofilm in the Bil gel + Laser (15 min) group, indicating a significant reduction in viable bacterial counts compared to the control (Fig. 7h). Notably, the treatment with Bil gel + Laser (15 min) also disrupted the dense architecture and thickness of the biofilm, as evidenced by its uneven surface and increased presence of open cavities. Statistical analysis of the data further corroborated these findings (Fig. 7i and j).

Collectively, Our findings demonstrate that the laser-irradiated composite hydrogel generates reactive oxygen species, which exert potent antibacterial effects by disrupting bacterial membrane integrity, thereby effectively eliminating susceptible bacteria while simultaneously inhibiting biofilm formation (Fig. 7k). Thereby, it emerges as a highly promising therapeutic candidate for the prophylaxis of wound infections and the facilitation of tissue regeneration.

### 2.9 In vivo wound healing in diabetic mice with infected wounds

Diabetic foot ulcer (DFU), a major complication in diabetic patients, exhibits impaired self-healing due to susceptibility to bacterial infection, inflammatory cell infiltration, compromised angiogenesis, and restricted cellular proliferation/migration [55]. Building on previous findings demonstrating the in vitro wound-healing potential of Bil hydrogels, we further evaluated the regenerative efficacy of microfiber-integrated Bil composite hydrogels using a full-thickness skin defect model in diabetic C57BL/6 mice.

**Fig. 8:**
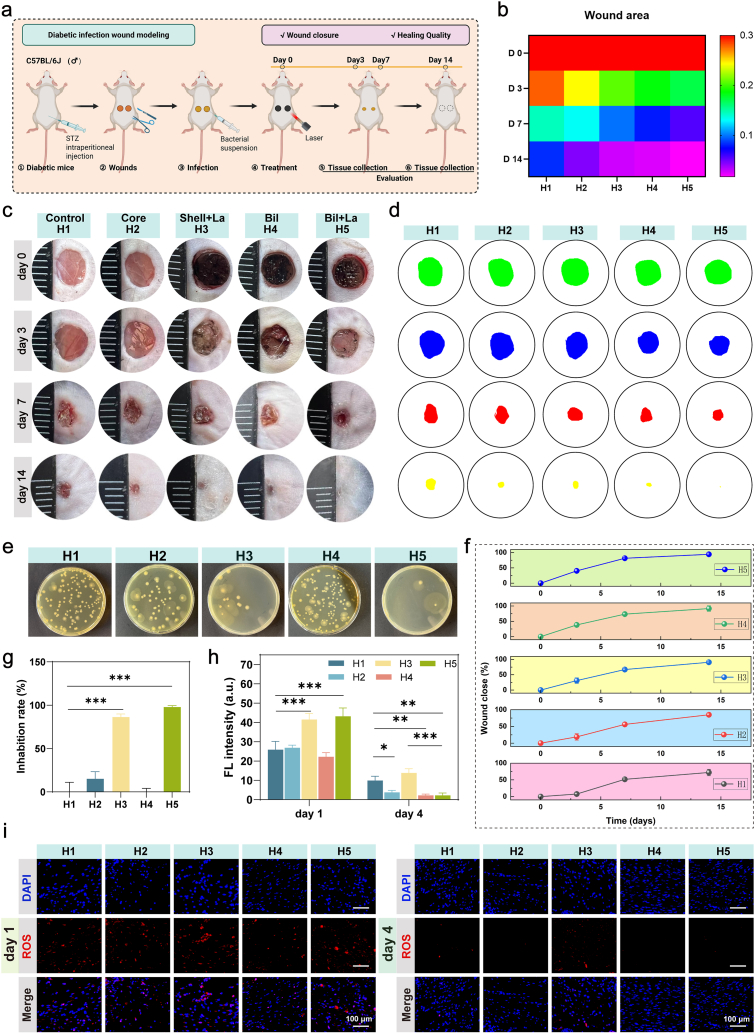
**The Bil Platform Accelerates Diabetic Infected Wound Healing In Vivo.** (a) Schematic diagram of the experimental timeline for establishing and treating full-thickness diabetic wound models. (b) Statistical heatmap of wound healing rates. (c) Representative photographic images of wounds in mice receiving different treatments from day 0 to day 14. (d) Simulated wound area profiles across various treatment groups. (e)) Images of *S. aureus* and *E.coli* colonies from the wound tissues on day 3 cultured on mannitol salt agar medium plates. (f) Quantification of wound closure rates over time (n = 3 independent experiments). (g) Statistical analysis of wound antibacterial rate data (n = 3 independent experiments). (h) Statistical analysis of DHE fluorescence intensity in wound tissue on days 1 and 4 (n = 3 independent experiments). (i) Representative fluorescence images of ROS-scavenging effects in wound tissues on day 1 and day 4. Nuclei are stained with DAPI (blue), and ROS levels are indicated by DHE fluorescence (red). The results are shown as the mean ± standard deviation (SD). The symbols ∗p < 0.05, ∗∗p < 0.01, ∗∗∗p < 0.001.

The experimental workflow (Fig. 8a) involved streptozotocin-induced diabetes confirmed by sustained hyperglycemia (random blood glucose ≥16.7 mmol/L) and polyuria/polydipsia symptoms. Dorsal full-thickness wounds (6 mm diameter) were treated with PBS (H1), Core (H2), Shell + laser irradiation (15 min, H3), Bil (H4), or Bil + laser irradiation (15 min, H5). All groups showed no signs of erythema, irritation, or infection. Macroscopic evaluation revealed accelerated wound closure in the Bil + laser group (H5) (Fig. 8b and c), achieving 81.42 ± 2.82% healing by day 7 versus 51.77 ± 3.14% (H1), 56.16 ± 6.08% (H2), 66.97 ± 3.94% (H3), and 73.37 ± 4.61% (H4). Complete re-epithelialization occurred by day 14 in H5, whereas residual wounds persisted in other groups (Fig. 8d and f). Notably, Bil alone (H4) outperformed individual Core (H2) or Shell (H3) components, confirming their synergistic action. By homogenizing the skin tissue surrounding the wounds harvested on day 3, we evaluated the anti-biofilm efficacy (Fig. 8e and g). Wounds treated with Bil under laser irradiation exhibited the lowest bacterial colony counts, achieving an inhibition rate of 97.85%, which indicates effective suppression of bacterial biofilm formation.

Quantitative DHE analysis revealed distinct spatiotemporal patterns of superoxide anion (O_2_^−^) generation throughout the healing process. At day 1 post-infection, both shell + laser and Bil + laser groupsexhibited significantly elevated fluorescence compared to non-irradiated controls, demonstrating verteporfin's (VP) potent photodynamic ROS generation during acute inflammation. Notably, by day 4, the dual-gel + laser group demonstrated markedly reduced DHE fluorescence versus the shell + laser group (Fig. 8h and i). These results establish that the biphasic system achieves dynamic ROS modulation: VP maintains robust photodynamic activity during infection while EGCG provides antioxidant protection during proliferative phase, creating a microenvironment that adapts to wound healing requirements.

**Fig. 9:**
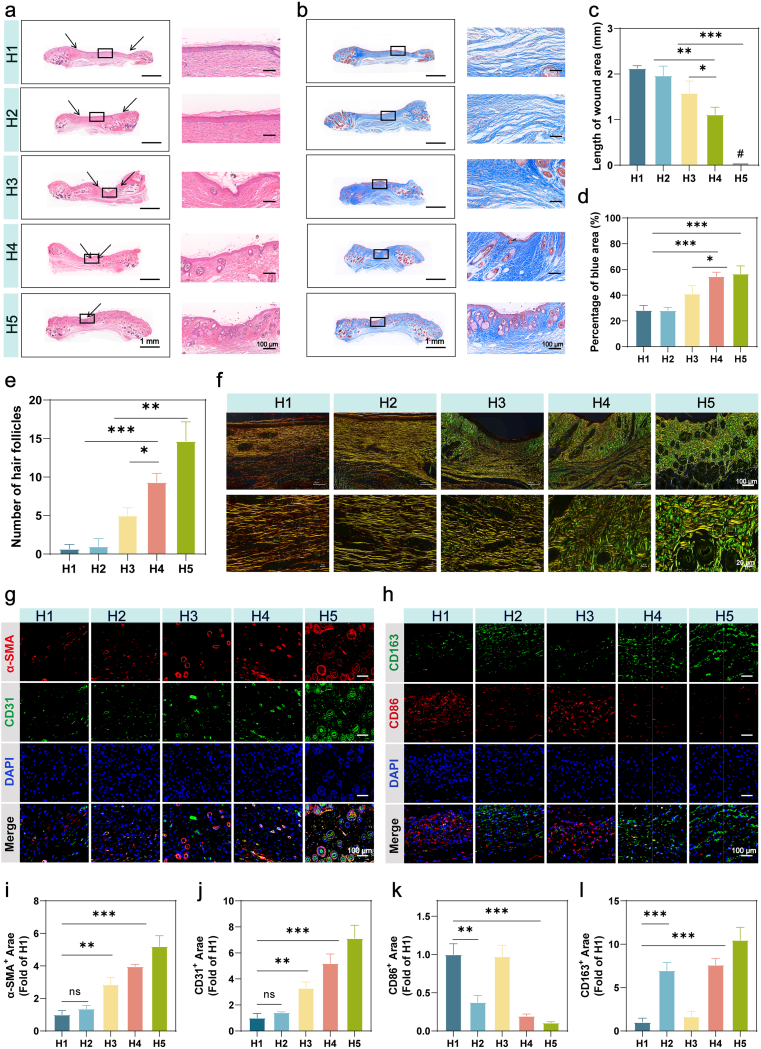
**The Bil composite hydrogel accelerates diabetic wound healing by enhancing granulation tissue formation, collagen deposition, angiogenesis, and anti-inflammatory responses.** (a) Histological analysis of wound tissues on day 14 via H&E staining. (b) Histological evaluation of collagen organization in wound sections on day 14 using Masson's trichrome staining. (c) Statistical analysis of wound contraction length (n = 3 independent samples). (d) Quantitative assessment of collagen deposition in the wound bed (n = 3 independent samples). (e) Statistical evaluation of hair follicle regeneration within the healed wound area (n = 3 independent samples). (f) Collagen architecture and maturity analysis in day 14 wounds via Picrosirius red staining. (g) Immunofluorescence staining of CD31 (endothelial cell marker) and α-SMA (mature vessel marker) in wound sections on day 14. (h) Immunofluorescence staining of M1 macrophage marker CD86 and M2 macrophage marker CD163 in wound tissues on day 14. (i, j) Quantitative analysis of α-SMA and CD31 expression levels in wound areas (n = 3 independent samples). (k, l) Quantitative analysis of CD86^+^ (M1) and CD163^+^ (M2) macrophage infiltration (n = 3 independent samples). The results are shown as the mean ± standard deviation (SD). The symbols ∗p < 0.05, ∗∗p < 0.01, ∗∗∗p < 0.001.

Histopathological evaluation of Bil microfiber composite hydrogels at the tissue level was performed using hematoxylin and eosin (H&E) staining on day 14 (Fig. 9a). While control, Core-only, and Shell-only groups exhibited localized deposition of immature granulation tissue at wound peripheries, the Bil + laser group demonstrated near-complete dermal regeneration with highly organized granulation tissue architecture. Quantitative morphometric analysis revealed significantly promotes wound closure in Bil + laser-treated wounds compared to PBS controls (1.5-fold increase), accompanied by enhanced epidermal layer integrity and reduced residual wound area relative to all comparator groups (Fig. 9c). This coordinated tissue restoration—spanning dermal matrix reorganization and epidermal barrier reformation—validates Bil's capacity to synchronize multi-layered regenerative processes, surpassing the partial healing outcomes achieved by individual core or shell components. Collagen deposition patterns in wound healing areas were systematically evaluated using Masson's trichrome staining. While all experimental groups exhibited increased collagen content compared to the blank control, the Bil + laser group demonstrated superior collagen deposition quality at day 14, characterized by densely packed, well-aligned collagen bundles with preferred orientation (Fig. 9b and d). This organized matrix architecture contrasted sharply with the disorganized, low-density collagen networks observed in other treatment groups. Furthermore, histomorphometric analysis revealed a significant increase in hair follicle neogenesis within the Bil + laser treatment sites compared to other groups (Fig. 9e), indicating enhanced regenerative capacity beyond basic wound closure. The coordinated improvement in both collagen maturation and appendage regeneration underscores the dual functionality of laser-activated Bil hydrogels in restoring structural and functional skin integrity.

Sirius red polarization microscopy (Fig. 9f) evaluated collagen remodeling, showing elevated type III collagen (green birefringence) and reduced type I collagen (yellow/orange) ratios in Bil-treated groups. The Bil + laser group exhibited the highest type III/I collagen ratio, indicative of scar-minimizing regenerative repair. These results collectively validate the hierarchical ROS modulation-driven therapeutic advantages of the core-shell Bil hydrogel system for diabetic wound management.

Neovascularization, critical for tissue survival and remodeling, was assessed via CD31 (endothelial marker) and α-smooth muscle actin (α-SMA, myofibroblast marker) immunostaining. While the PBS-treated group (H1) showed sparse, immature vasculature, robust angiogenesis with well-structured capillaries was observed in H4 and H5, evidenced by intense α-SMA positivity and linear CD31^+^ vessel patterning (Fig. 9g–i and j). Cellular proliferation, a key determinant of re-epithelialization efficacy, was analyzed through Ki67 staining. The Bil + laser group demonstrated markedly higher proliferation rates than controls (Fig. S7a and b), correlating with accelerated wound closure. Compromised proliferative capacity, as seen in chronic wounds, often leads to delayed healing and pathological scarring [56,57].

Finally, to evaluate inflammatory responses at wound sites, immunofluorescence staining was performed to quantify CD86 (M1 macrophage marker) and CD163 (M2 macrophage marker) expression. Intriguingly, the Bil + laser group (H5) exhibited significantly reduced CD86-positive signals alongside enhanced CD163 expression (Fig. 9h–k and l), indicating a preferential shift toward pro-regenerative M2 macrophage polarization. Collectively, these findings underscore the Bil hydrogel's multifunctional therapeutic benefits: (1) immunomodulation through macrophage phenotype switching, (2) structural reinforcement via organized neovascularization, and (3) activation of resident cell proliferation. This tripartite mechanism synergistically drives scar-suppressed regeneration in diabetic wounds.

### 2.10 Wound healing mechanisms of the microfiber composite hydrogel

**Fig. 10:**
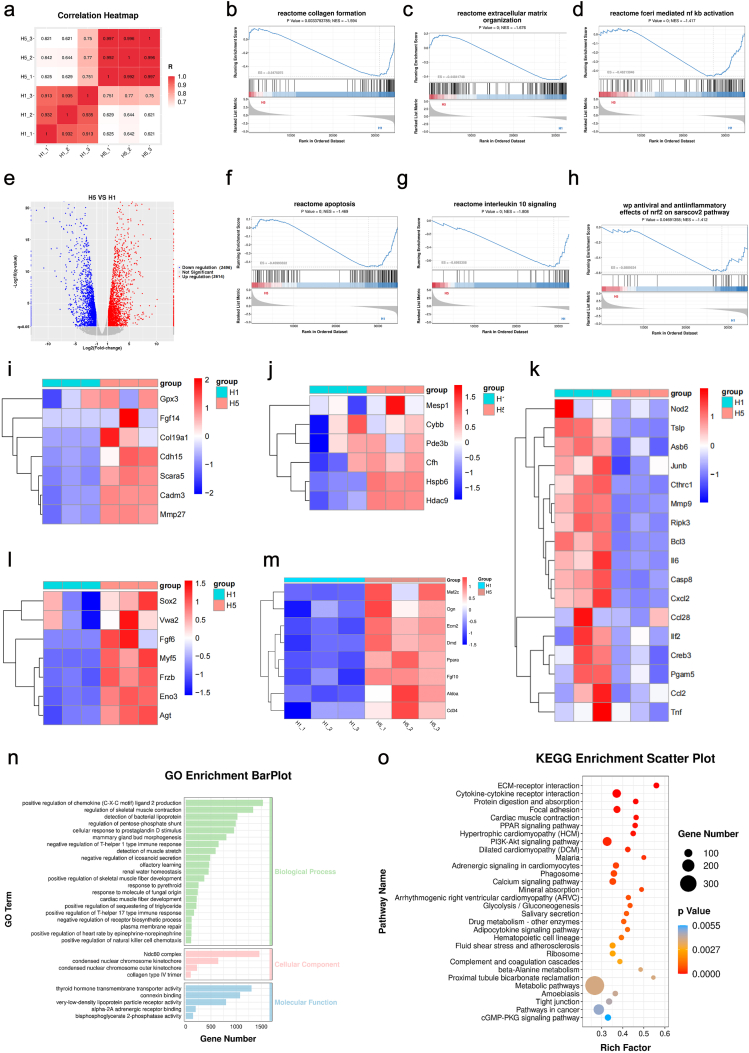
**Transcriptome Sequencing Analysis of Wound Tissues.** (a) Correlation analysis between biological replicates of the Control and Bil + La groups. (b) Volcano plot displaying upregulated and downregulated genes in wound sites of the Bil + La group compared to the Control group. (c–h) Gene Set Enrichment Analysis (GSEA) of biological processes affected by Bil + La treatment. (i) Significantly differentially expressed genes related to wound healing after Bil + La hydrogel treatment. (j) Significantly differentially expressed genes associated with angiogenesis following Bil + La hydrogel treatment. (k) Significantly differentially expressed genes implicated in tissue regeneration in the Bil + La group. (l) Significantly differentially expressed genes linked to collagen remodeling after Bil + La treatment. (m) Significantly differentially expressed genes involved in skin appendage regeneration. (n) Gene Ontology (GO) enrichment analysis of differentially expressed genes. (o) KEGG pathway enrichment analysis of differentially expressed genes.

Our findings establish Bil-based hydrogels as a transformative therapeutic strategy for diabetic wound management, demonstrating spatiotemporal control over three critical healing phases: oxidative stress modulation through programmable ROS generation/scavenging, inflammation resolution via macrophage phenotype switching, and tissue regeneration via matrix remodeling. RNA sequencing analysis of day 14 wound tissues provided critical mechanistic insights. Initial correlation assessment between the control (H1, PBS-treated) and Bil + laser (H5) groups demonstrated strong system consistency (Fig. 10a). While 13,530 genes showed conserved expression patterns, we identified 2614 significantly upregulated and 2496 downregulated genes in the H5 group versus controls (Fig. 10b). This distinct differential expression signature demonstrates the remarkable capacity of Bil technology to reprogram healing pathways. Differential expression analysis revealed 2614 upregulated genes in the H5 model, including wound healing-associated genes (e.g., Gpx3, Fgf14, Col19a1, Cdh15, Scara5, Cadm3, Mmp27) (Fig. 10i), key regulators of vascular network maturation (e.g., Mesp1, Cybb, Pde3b, Cfh, Hspb6, Hdac9) (Fig. 10j), and tissue-regenerative effectors promoting epidermal-dermal crosstalk (e.g., Sox2, Fgf6, Vwa2, Myf5, Eno3, Frzb, Agt) (Fig. 10l). Notably, genes regulating appendage regeneration (e.g., Mef2c, Ecm2, Ogn, Dmd, Ppara, Fgf10, Aldoa, CD34) (Fig. 10m) and matrix metalloproteinases (Mmp27) showed selective activation, consistent with histological observations of hair follicle neogenesis and organized collagen deposition. Conversely, 2496 downregulated genes included fibrosis-related genes (e.g., Runx1, Acan, Actg1, Gm4430) (Fig. S6c) and pro-inflammatory mediators (e.g., Tslp, Ccl28, Asb6, Ilf2, Bcl3, Casp8, Junb, Mmp9, Nod2, Pgam5, Ripk3, Tnf, Traf5, Il6) (Fig. 10k), whose suppressed expression aligned with immunohistochemical findings showing reduced CD86^+^ M1 macrophages and increased CD163+ M2 cell populations.

Kyoto Encyclopedia of Genes and Genomes (KEGG) pathway analysis demonstrated marked upregulation of multiple pathways related to inflammatory response, oxidative stress, collagen fibril organization, and extracellular matrix remodeling (Fig. 10n). Gene Ontology (GO) enrichment analysis revealed that Bil + La treatment significantly upregulated wound healing-associated genes involved in angiogenesis, cell migration, collagen deposition, and extracellular matrix organization (Fig. 10o). To further delineate the underlying signaling mechanisms, we performed Gene Set Enrichment Analysis (GSEA), which identified the critical involvement of the 'Antiviral and anti-inflammatory effects of Nrf2 on SARS-CoV-2′ pathway, IL-10 signaling, apoptosis, collagen formation, extracellular matrix organization, and NF-κB signaling pathways in Bil hydrogel-mediated wound repair (Fig. 10c–h). Notably, the NF-κB pathway—a master regulator of inflammatory responses governing multiple cytokine cascades—was significantly suppressed in H5-treated groups. This inhibition mechanistically aligns with observed reductions in pro-inflammatory M1 macrophage infiltration and concomitant increases in pro-regenerative M2 populations, suggesting Bil hydrogel orchestrates macrophage polarization through NF-κB pathway modulation. Building on these findings, we propose that Bil hydrogel mitigates pathological inflammation by intercepting NF-κB-driven transcriptional activation, thereby disrupting the self-perpetuating inflammatory loops characteristic of diabetic wounds. This anti-inflammatory reprogramming synergizes with the material's pro-regenerative functions to create an immunologically balanced microenvironment conducive to scar-free healing. Future investigations will employ NF-κB pathway-specific inhibitors/activators and chromatin accessibility assays to delineate its centrality to Bil's therapeutic efficacy, with the ultimate goal of engineering precision-controlled hydrogel systems for pathway-targeted intervention.

### 2.11 Microfiber composite hydrogel promotes scar-free healing

**Fig. 11:**
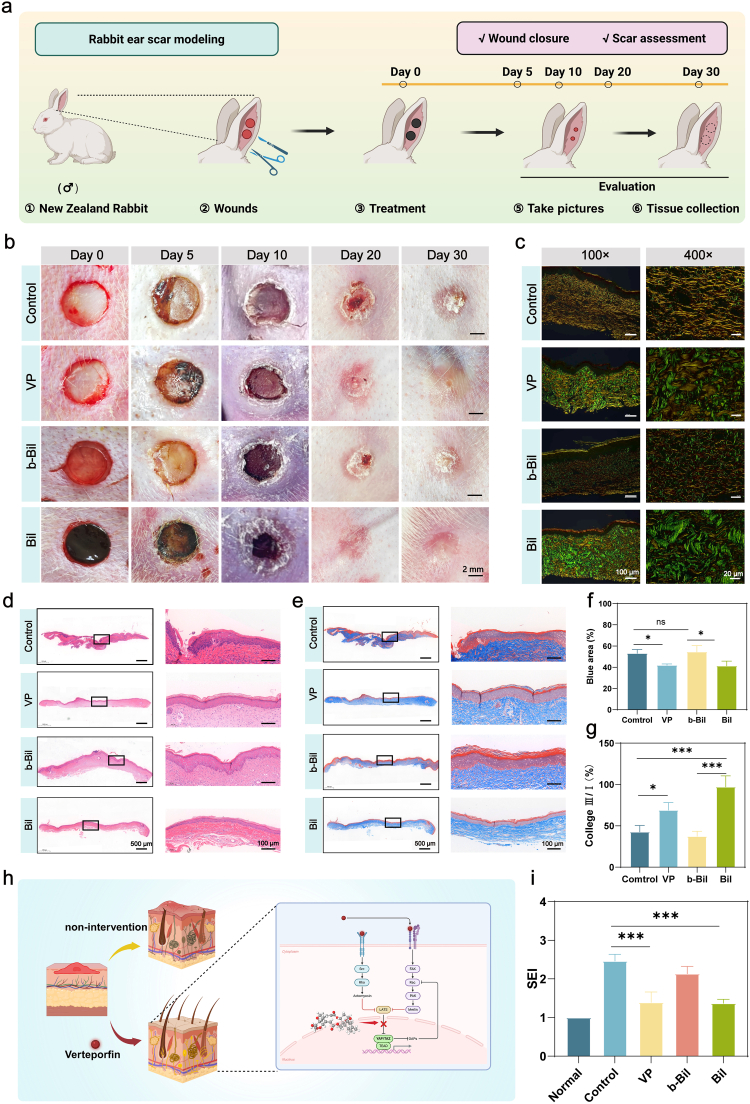
**Bil Inhibits Scar Formation in a Rabbit Ear Model.** (a) Schematic diagram of the experimental design for evaluating scarless wound healing following Bil treatment. (b) Representative photographs of rabbit ear wounds across different treatment groups at various time points. (c) Picrosirius red staining of wound sections on day 30. (d) H&E staining of wound tissues harvested on day 30. (e) Masson's trichrome staining of collagen deposition in day 30 wound sections. (f) Statistical analysis of collagen density in healed wound tissues (n = 3 independent samples). (g) Quantitative analysis of the collagen type III to type I ratio in the wound tissue (n = 3 independent samples). (h) Proposed mechanism of verteporfin-mediated inhibition of scar formation. (i) Scar elevation index (SEI) quantification across experimental groups (n = 3 independent samples). The results are shown as the mean ± standard deviation (SD). The symbols ∗p < 0.05, ∗∗p < 0.01, ∗∗∗p < 0.001.

To address the limitations of murine dorsal skin models (which lack mechanical tension for fibrotic scar formation), we employed a validated rabbit ear hypertrophic scar model that mimics human healing dynamics through high mechanical stress and scar-prone repair processes [58]. The schematic diagram in Fig. 11a depicts the experimental workflow. Adult New Zealand white rabbits were randomized into four groups: PBS control, VP solution, blank Bil hydrogel (VP-free, b-Bil), and VP-loaded Bil hydrogel (Bil). Macroscopic evaluation at day 30 revealed distinct morphological outcomes—VP solution and VP-loaded Bil groups exhibited flat, pale scars compared to the elevated, erythematous scar tissue observed in PBS and blank Bil groups (Fig. 11b). Quantitative analysis demonstrated significantly reduced scar elevation index (SEI) in VP-containing treatments versus controls (Fig. 11i), a finding histologically corroborated by marked decreases in scar thickness in VP-loaded Bil specimens using Masson's trichrome and H&E staining (Fig. 11d and e). Furthermore, Masson's trichrome staining demonstrated a marked decrease in collagen fiber (blue) deposition in skin tissues treated with VP solution and Bil + VP compared to the other two experimental groups (Fig. 11f).

Sirius red polarization microscopy further revealed enhanced type III collagen deposition (green birefringence) and improved collagen III/I ratios in VP-treated groups, indicative of regenerative matrix remodeling(Fig. 11c and g). Recent studies have indicated that Engrailed-1 lineage-positive fibroblasts (EPFs) play a critical role in skin scar formation, while suppressing YAP via VP-mediated inhibition to block En1 activation can promote scarless skin regeneration [59]. Notably, while both blank Bil and VP-loaded Bil accelerated wound closure, only the VP-incorporated formulation achieved concurrent scar suppression through mechanochemical regulation of fibroblast activity (Fig. 11h). This finding is consistent with previously published reports. This differential outcome underscores VP's critical role in disrupting fibrotic cascades while maintaining Bil's intrinsic healing-promoting properties. The synergistic combination of VP's anti-fibrotic action with Bil's structural guidance establishes this composite system as a dual-functional platform capable of addressing both wound closure and aesthetic outcomes in tension-prone healing environments.

## 3 Conclusion

The physiological wound healing process consists of four sequential and overlapping phases: hemostasis, inflammation, proliferation, and remodeling. Under pathological conditions such as diabetes, severe infection, or chronic ischemia, however, this process often stagnates in a prolonged inflammatory phase, characterized by bacterial biofilm formation, dysregulated growth factor expression, impaired angiogenesis, and abnormal extracellular matrix deposition, leading to non-healing chronic wounds. Currently, biomaterials represented by hydrogels, nanofibers, and antibacterial dressings primarily promote healing by providing a moist environment, controlling infection, or delivering single growth factors. Nevertheless, most existing systems suffer from major limitations: poor dynamic responsiveness, failing to adapt intelligently to the varying demands of different healing stages; limited capacity for coordinated regulation of the complex pathological microenvironment (e.g., high oxidative stress, immune dysregulation); and difficulty in precisely orchestrating multicellular behaviors and sequential vascularization processes. Consequently, there remains a substantial gap in achieving functional tissue regeneration.

Inspired by the three-dimensional steel-concrete composite structures in civil engineering, we have developed a bionic core-shell system (denoted as Bil) for diabetic wound management through the hierarchical integration of a nanofiber-reinforced “steel” core and a reactive oxygen species (ROS)-responsive “concrete” shell. This hierarchical architecture comprises two functionally integrated zones: (1) A ROS-responsive “concrete” matrix constructed via TSPBA-crosslinked poly(vinyl alcohol), which simultaneously encapsulates human umbilical cord mesenchymal stem cell-derived exosomes (hUC-MSCs-Exos) and verteporfin (VP). This compartment exhibits photodynamic antibacterial capability under laser irradiation while enabling controlled release of exosomes synchronized with the demands of the cell proliferation phase. (2) A mechanically enhanced “steel” core fabricated through electrospinning of sericin composite fibers, which establishes a long-term antioxidant reservoir enriched with epigallocatechin gallate (EGCG) for inflammation resolution. In a preclinical diabetic wound model, Bil demonstrated triphasic therapeutic precision through material-guided microenvironment reprogramming: the initial phase employed VP-mediated ROS generation to eradicate pathogenic biofilms, followed by EGCG-driven suppression of oxidative stress and facilitation of macrophage repolarization toward a regenerative M2 phenotype. Concurrently, the sustained release of hUC-MSCs-Exos not only enhanced the biological functions of fibroblasts and vascular endothelial cells, but also—through VP-mediated inhibition of the YAP signaling pathway—effectively blocked the transition of fibroblasts into myofibroblasts. This spatiotemporally programmed treatment modality successfully resolves the pathophysiological conflict between inflammation and proliferation in chronic wounds, as evidenced by accelerated re-epithelialization, enhanced angiogenesis, remodeled collagen matrix, and reduced fibrotic scarring—collectively underscoring its clinical translational value. Compared to prior research [11,60], The present hydrogel system exhibited favorable inflammatory modulation and collagen remodeling capability.Notably, the wound closure rate reached 81.42% at day 7, which was higher than those reported in Ref. [11] (76.46%) and Ref. [60] (80%).Meanwhile, collagen deposition at day 14 reached 56.59%, exceeding the 53% reported in Ref. [60]. These results demonstrate the distinct advantages of our sequential-responsive design in promoting efficient wound healing and tissue remodeling. This innovatively designed biomimetic strategy establishes a novel paradigm for intelligent wound management. By synergistically integrating structural and mechanical cues through material engineering, it enables coordinated regulation across sequential healing stages—spanning reactive oxygen species homeostasis, antimicrobial defense, immunomodulation, and extracellular matrix remodeling.

Based on the above results, we propose an integrated and testable mechanistic model that links material properties, ROS dynamics, cellular behavior, and in vivo scar outcomes.Spatio-temporally regulated ROS generation and scavenging modulated the intracellular redox environment, which may further influence macrophage polarization through redox-sensitive upstream regulators that are functionally linked to the NF-κB pathway, consistent with our transcriptomic analysis.

Meanwhile, VP-mediated inhibition of YAP signaling may suppress fibroblast activation, myofibroblast formation, and excessive extracellular matrix deposition, which directly contributes to attenuated scar formation in the rabbit model.This sequential and coordinated regulatory cascade connects material design, immune modulation, and fibrotic responses to promote scarless wound healing, and provides a mechanistic foundation for the rational design of smart wound repair materials.

Despite the promising results, several limitations must be acknowledged. Future research should systematically elucidate the precise molecular and cellular mechanisms by which the composite hydrogel promotes healing and modulates scarring. Specifically, in vivo tracking of key signaling pathways, single-cell transcriptomic analysis of healing niches, and functional validation of candidate targets are needed to replace current speculative interpretations with mechanistic evidence.

## CRediT authorship contribution statement

**Pan Du:** Conceptualization, Formal analysis, Methodology, Software, Writing – original draft, Writing – review & editing. **Jin Li:** Data curation, Formal analysis, Methodology, Software. **Jun Pu:** Formal analysis, Methodology, Software. **Shuqian Dou:** Data curation, Methodology. **Gaofei Zhang:** Formal analysis, Resources. **Kongjia Wu:** Software, Validation. **Shihao Deng:** Methodology. **Qiulei Wang:** Methodology. **Wenjun Liu:** Funding acquisition, Investigation, Project administration, Supervision.

## Declaration of competing interest

All the authors declare no conflict of interest.

## Figures and Tables

**figs1:**
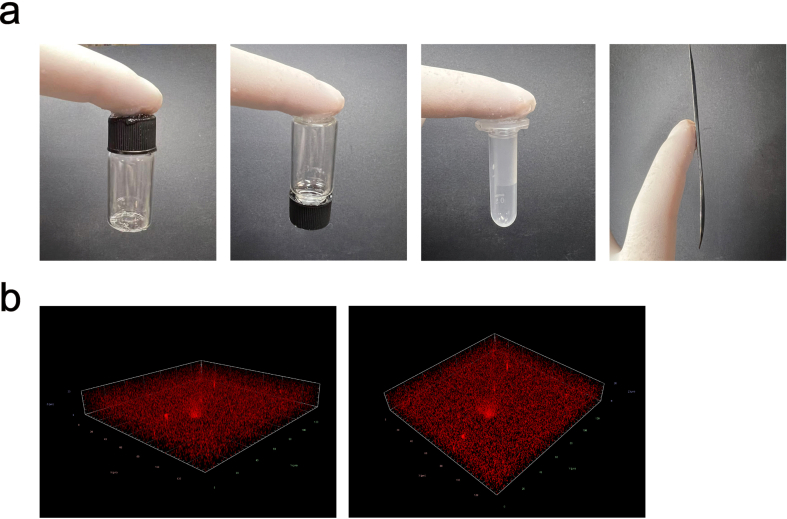


## Data Availability

Data will be made available on request.
