# Supplementary material for: Smart ROS-regulating biomimetic hydrogel promotes scarless diabetic wound healing via macrophage reprogramming
Source: Mater Today Bio. 2026 Mar 27;38:103061. doi: 10.1016/j.mtbio.2026.103061 (PMC13087777; doi:10.1016/j.mtbio.2026.103061)
Supplement: Multimedia component 1 [file mmc1.docx]

Supporting information

**Smart ROS-Regulating Biomimetic Hydrogel Promotes Scarless Diabetic Wound Healing via Macrophage Reprogramming**

1. **Materials and methods**

**1.1. Materials**

Polyvinyl alcohol (PVA), Epigallocatechin gallate (EGCG), Verteporfin (VP), tetrahydrofuran (THF), tetrahydrofuran, sodium salicylatewere obtained from Macklin (China). Lipopolysaccharide (LPS), poly(lactic-co-glycolic acid (PLGA), Dichloromethane were purchased from Sigma-Aldrich (USA). interleukin 4 (IL 4), interleukin 13 (IL 13), Interferon-gamma (IFN-γ) were obtained from Peprotech (USA). Beyoclick EDU-555 cell proliferation detection kit, CCK8 test kit, Reactive oxygen species detection kit, Total antioxidant capacity testing kit (ABTS), DPPH radical scavenging assay kit, Dihydroethidium, Live/Dead Assay Kit were purchased from Beyotime (China). Anti-VEGF antibody, Anti-CD 206 antibody, Anti-CD 86 antibody, Anti-CD 163 antibody, Anti-CD 31 antibody, Anti α-smooth muscle actin antibod and KI 67 antibody were purchased from Abcam (USA). Anti-type I, III collagen were purchased from proteintech Proteintech Group, Inc (China). AntibodyAnti-Tsg101, Anti-calnexin were purchased from huabio.Glycerol, streptozocin (STZ) were purchased from Solarbio (China). Tribromoethanol were obtained from Nanjing Aibei Biotechnology Co., Ltd (China). Rhodamine-phalloidin, FITC affinipure goat anti rabbit, Rhodamine affinipure goat anti mouse were purchased from Yeasen (China). Matrigel was purchased from Corning (356231, USA). N,N,N',N'-Tetramethyl-1,3-propanediamine (TMPDA), 4-(bromomethyl)phenylboronic acid (4-BPBA), N,N-Dimethylformamide (DMF) were purchased from Aladdin (China).

**1.2. Isolation and Identification of hUC-MSC-Exos**

hUC-MSCs-derived exosomes (hUC-MSCs-Exos) were isolated from conditioned media via ultracentrifugation as described[1,2]. Briefly, hUC-MSCs were cultured in DMEM supplemented with exosome-depleted FBS. Upon reaching 80-90% confluency, media were collected, centrifuged (10000 g, 30 min) to remove cellular debris, and filtered through 0.22 μm membranes. Exosomes were pelleted by ultracentrifugation (100000g, 90 min), washed with PBS via repeat ultracentrifugation, and stored at -80°C.Exosome quantification was performed using a BCA assay. Size distribution were analyzed by dynamic light scattering (DLS; Malvern Instruments, UK), while ultrastructure was characterized via TEM after uranyl acetate staining. Western blot confirmed expression of exosomal marker TSG101 and absence of cytoplasmic contaminant calnexin. A study on the phagocytosis of Exos encapsulated within the shell-layer hydrogel by fibroblasts and vascular endothelial cells was conducted as follows. First, Exos were isolated, characterized, and labeled. The Exos were initially stained with PKH 26, followed by co‑incubation with cells for 24 hours. The cells were then fixed at room temperature and stained with TRITC‑phalloidin and 4′,6‑diamidino‑2‑phenylindole (DAPI) to visualize the cytoskeleton and nucleus, respectively. Finally, the labeled cells were observed using a confocal microscope.

**1.3. Synthesis of PLGA-Exos**

PLGA-Exos nanoparticles were synthesized using a double emulsion (W/O/W) solvent evaporation method [3]. Briefly, PLGA (50 mg/mL in dichloromethane) was emulsified with aqueous hUC-MSCs-Exos (10μg/mg PLGA) by homogenization (5000 rpm, 10 s, ice bath) to form a primary W/O emulsion. This emulsion was immediately dispersed into 2% PVA solution, homogenized (10000 rpm, 10 s), and sonicated (50% amplitude, 5 s on/off cycles, 60 s) to generate W/O/W droplets. After solvent removal via 5 h magnetic stirring in ultrapure water, nanoparticles were collected by centrifugation (5000 rpm, 15 min), washed thrice, and lyophilized after overnight cryopreservation at -20°C.

**1.4. Preparation of shell**

TSPBA was synthesized following an adapted quaternization protocol [4]. Briefly, N,N,N',N'-tetramethyl-1,3-propanediamine (TMPDA, 0.2 g) was dissolved in anhydrous DMF (40 mL) under heating (60℃). To this solution, 4-(bromomethyl)phenylboronic acid (4-BPBA, 1.0 g) was added sequentially, and the reaction proceeded at 60℃ for 24 h with vigorous stirring. The crude product was precipitated in tetrahydrofuran (THF, 100 mL), filtered, and washed thrice with THF to eliminate unreacted precursors. After freeze-drying, *N^1^*-(4-boronobenzyl)-*N^3^*-(4-boronophenyl)-*N^1^*, *N^1^*, *N^3^*, *N^3^*tetramethyl-1,3-propanediaminium (TSPBA) was obtained as a white crystalline solid (0.6 g). A ROS-responsive hydrogel precursor was formulated by blending polyvinyl alcohol (PVA, 2 wt%) with TSPBA (2 wt%) at a 2:1 mass ratio to yield 2 mL of homogeneous mixture. The precursor was cured under ambient conditions for 12 h to achieve structural integrity. The PVA-TSPBA hydrogel was subsequently functionalized through incorporation of verteporfin (VP, 60 μg) and 20 mg of PLGA-encapsulated exosome microspheres (PLGA-Exos), yielding a drug-eluting shell matrix capable of on-demand therapeutic release.

**1.5. Preparation of core and Bil**

Silk fibroin (SF) was extracted from Bombyx mori cocoons via an optimized degumming-dialysis protocol [5]. Briefly, 30 g cocoons were boiled in 0.02 M Na_2_CO_3_ (14 L) at 100℃ for 30 min to remove sericin, followed by rinsing, drying, and dissolution in 9.3 M LiBr (60℃, 5 h). The solution underwent 72 h dialysis (MWCO 12 kDa) against ultrapure water, with contaminants removed through dual centrifugation cycles (10000 g, 4℃, 20 min). Purified SF was lyophilized (48 h) and stored at -20℃.

SF-Gel nanofibrous membranes were fabricated via electrohydrodynamic spinning. A precursor solution containing SF (7 wt%) and gelatin (3 wt%) in formic acid was homogenized with epigallocatechin gallate (EGCG, 700 μg/mL) under 2 h magnetic stirring. The homogenized solution was loaded into a 5 mL syringe equipped with a 21G blunt needle and electrospun (8 kV, 12 cm collector distance, 5 mL/h flow rate). Nanofibers collected on aluminum foil were solvent-annealed in a fume hood (72 h) to ensure complete formic acid evaporation.

The bilayer system (Bil) was synthesized through a sequential templating strategy. First, a SF-Gel nanofibrous membrane was suspended within a polydimethylsiloxane (PDMS) mold, followed by infusion of PVA-TSPBA precursor solution (2 wt%) to fully encapsulate the nanofiber scaffold. Ambient drying (25℃, 12 h) induced interfacial crosslinking, yielding a microfiber-reinforced Bil hydrogel with hierarchical architecture. To enhance the hydration capacity of the Bil hydrogel, the composite was immersed in glycerol (99.8%) for 5 h, yielding the glycerol-plasticized Bil-Gly system through hygroscopic modification.

**1.6. Characterization of Bil Microfiber Hydrogel**

The core-shell microstructure and PLGA microspheres were characterized via scanning electron microscopy (SEM) following standardized sample preparation. Cryo-fixed specimens were lyophilized for 48 hr at -50℃ under 0.1 mbar vacuum, then sputter-coated with a 5-10 nm gold layer (15 mA, 60 s) using a magnetron coater to ensure surface conductivity prior to SEM imaging at 5 kV accelerating voltage.

Hydrogel specimens were fabricated into cuboid geometries for mechanical characterization. Uniaxial tensile testing was performed using a servo-controlled testing system (SFMIT-1000N, China) equipped with a 1000 N load cell. The mechanical responses of Bil microfiber-reinforced hydrogels - prepared with varied electrospinning durations (30s-8 min) and freeze-thaw cycles (0-4 cycles) - were evaluated at a constant crosshead speed of 2 mm/min. All mechanical parameters are reported as mean ± SD (n = 3 independent batches).

**1.7. ROS-responsive behavior of shell**

To evaluate the ROS-responsive degradation behavior of the shell matrices, specimens were incubated in PBS (control) and PBS containing hydrogen peroxide (H_2_O_2_) at concentrations of 0.1, 0.5, 1, and 2 mM. Gravimetric analysis was performed at predetermined intervals (1, 5, 10, 20, 30, 48, and 72 h) by carefully blotting surface moisture and measuring mass retention (±0.1 mg precision) to quantify oxidative erosion kinetics. Parallelly, Bil composites co-loaded with verteporfin and exosomes were immersed in PBS and PBS supplemented with 1 mM H_2_O_2_, then incubated at 37°C under orbital agitation (400 rpm) to simulate physiological and oxidative microenvironments. At specified timepoints (VP: 1–72 h; Exos: 1–20 d), 200 μL aliquots were collected and replaced with fresh medium to maintain sink conditions. VP release profiles were determined via UV-vis spectroscopy by monitoring absorbance at 689 nm, while Exos release kinetics were analyzed using bicinchoninic acid technique (BCA).

**1.8 Drug release of EGCG**

The release profile of EGCG from the hydrogels was investigated using a direct immersion method.Briefly, 1 g of Bil hydrogel was immersed in 20 mL of 1 mM H₂O₂ solution and incubated in a shaker at 37 °C with a constant shaking speed of 120 rpm.At predetermined time intervals, 1 mL of the supernatant was collected, and an equal volume of pre‑warmed fresh PBS was replenished to maintain a constant volume.The concentration of released EGCG was quantified by the Folin‑Ciocalteu colorimetric method.In brief, 20 μL of the release medium was mixed with 100 μL of 10% (v/v) Folin‑Ciocalteu reagent in a 96‑well plate, followed by the addition of 80 μL of 7.5% (w/v) sodium carbonate solution.After incubation at room temperature in the dark for 60 min, the absorbance at 765 nm was measured using a microplate reader. A standard curve was established using EGCG standard solutions at known concentrations with the same Folin-Ciocalteu procedure. All experiments were performed in triplicate.

**1.9. Biocompatibility**

Hemocompatibility of the microfiber composites was evaluated via a standardized hemolysis assay using fresh goat blood. Material extracts were prepared by immersing core, shell, and Bil composite specimens in 1 mL saline (0.9% NaCl) at 37°C for 12 h. Subsequently, 20 μL anticoagulated whole blood was introduced to each extract and incubated under physiological conditions (37°C, 120 rpm orbital shaking) for 2 h. Post-incubation, suspensions were centrifuged at 200 × gfor 10 min, and hemoglobin release was quantified by measuring supernatant absorbance at 545 nm using a microplate reader (BioTek Synergy H1).Positive controls (100% hemolysis) consisted of 5% blood lysed in deionized water, while negative controls (0% hemolysis) contained 5% blood in saline. Hemolysis ratio (HR) was calculated as:

$Hemolysis rate (\%)=\frac{test sample- negative control}{positive control- negative control}$ × 100%

Additionally, erythrocyte morphology in each sample was examined under a light microscope.

Cytocompatibility of the materials was evaluated using a transwell co-culture system combined with metabolic activity (CCK-8) and live/dead cell viability assays. L929 fibroblasts were seeded in the lower chamber (5×10³ cells/well), while test samples were placed in the upper chamber. After incubation under physiological conditions (37℃, 5% CO₂) for 1, 2, and 3 d, CCK-8 reagent was added to the culture medium, and absorbance at 450 nm was quantified using a microplate reader (BioTek Synergy H1) to assess proliferative activity. For live/dead staining, cells were incubated with calcein-AM/propidium iodide (Thermo Fisher) in PBS for 30 min under light-protected conditions. Viable cells (green fluorescence, calcein-AM) and dead cells (red fluorescence, PI) were imaged via fluorescence microscopy (Olympus IX83).

Cytoskeletal architecture of L929 fibroblasts co-cultured with samples was analyzed via fluorescence staining. Cells were seeded in the lower Transwell chamber (5×10⁴ cells/well) and incubated for 3 d under standard culture conditions (37°C, 5% CO₂). Following fixation (4% paraformaldehyde, 15 min), filamentous actin was labeled with TRITC-phalloidin (1:200), while nuclei were counterstained with DAPI (1 μg/mL). images were acquired using laser scanning confocal microscopy (Leica TCS SP8). Three random fields per sample were quantified using ImageJ (FIJI distribution) to determine cell spreading area, expressed as mean ± SD (n=3 biological replicates).

To assess in vivo toxicity, 0.5 g of core, shell, and Bil were surgically implanted subcutaneously in rats. After 10 days, rats were executed , and main organs (heart, liver, spleen, lungs, kidneys and skin) were histologically analyzed (H&E staining) to determine the existence of possible organ damage.

**1.10. In vitro wound healing test**

A scratch wound healing assay was performed to evaluate L929 fibroblast and HUVECs migration. Cells were seeded in 6-well plates at 1 × 10⁶ cells/mL and cultured to confluence (24 h, 37°C, 5% CO₂). A standardized scratch was created in the monolayer using a sterile 200 μL pipette tip, followed by PBS washes to remove debris. Cell migration was monitored via live-cell imaging using an inverted fluorescence microscope (Olympus IX83) equipped with an environmental chamber (37°C, 5% CO₂). Images were captured at 0, 24, and 48 h post-scratch. Scratch healing rate (%) = (Co-Ct)/Co × 100%, where C0 and Ct represent the area of scratches before and after the intervention, respectively.

**1.11. Edu Assay**

The proliferation of different groups of L929 and HUVECs cells was assessed using the Beyoclick EDU-555 Cell Proliferation Kit. Specifically, after operating according to the manufacturer's protocol, cells from each group were photographed using an inverted fluorescence microscope. At least 3 fields of view were selected for each sample and the average percentage of EdU-positive cells was calculated using ImageJ.

**1.12. Tube Formation Assay**

The angiogenic potential of HUVECs was evaluated using a Matrigel-based tubule formation assay. Briefly, 50 μL growth factor-reduced Matrigel (Corning, 356231) was plated into pre-chilled 96-well plates and polymerized at 37°C (5% CO₂) for 1 h. HUVECs (4×10⁴ cells/well, Lonza) were seeded onto the gel-coated wells and allowed to form vascular networks for 6 h under standard culture conditions. Tubule formation was assessed via phase-contrast microscopy (Nikon Eclipse Ti2) at 10× magnification, with three random fields per well imaged.Quantitative angiogenesis parameters were analyzed using ImageJ (v1.53) with the Angiogenesis Analyzer plugin. Data were normalized to untreated controls and expressed as mean ± SD (n=3 independent experiments).

**1.13. Immunofluorescence assay**

RAW264.7 cells were seeded in 24-well plates at 5×10⁴ cells/well and cultured for 24 h (37°C, 5% CO₂). For M1 polarization, cells were co-stimulated with 200 ng/mL LPS and 2.5 ng/mL IFN-γ (serving as negative control). M2 polarization was induced using 20 ng/mL IL-4 and 20 ng/mL IL-13 (serving as positive control). Test groups included samples placed in Transwell upper chambers during stimulation. Untreated cells served as blank controls. Post-treatment, cell morphology was photographed and recorded, followed by fixation with 4% paraformaldehyde (15 min), permeabilization with 0.2% Triton X-100 (10 min), and blocking with 5% BSA (1 h).Immunostaining was performed using rabbit anti-CD86 (1:1000, M1 marker) and mouse anti-CD163 (1:1000, M2 marker) primary antibodies (4°C, overnight). After PBS washes, cells were incubated with FITC-conjugated goat anti-rabbit IgG (1:500) and rhodamine B-conjugated goat anti-mouse IgG (1:500) secondary antibodies (60 min, RT), followed by DAPI nuclear counterstaining (10 min). Fluorescence images were acquired using an inverted fluorescence microscope (Nikon Eclipse Ti2, 20× objective).

Collagen synthesis (type I/III) and angiogenic potential (VEGF) were quantified in vitro via immunofluorescence. For in vivo analyses, tissue sections were immunolabeled with CD86 (inflammatory M1), CD163 (anti-inflammatory M2), CD31 (vascularization), α-SMA (fibrosis), and Ki67 (proliferation). Quantitative histomorphometry was conducted using ImageJ image analysis software.

**1.14. In vitro antibacterial activity of Bil**

The antibacterial efficacy of the samples was evaluated against Gram-negative (Escherichia coli, *E. coli*) and Gram-positive (Staphylococcus aureus, *S. aureus*) bacteria through colony-forming unit (CFU) quantification and live/dead bacterial viability assays. The experimental groups were designated as follows: Control (equivalent volume of PBS added, G1), Laser (laser irradiation only, G2), Bil(no-VP)+Laser (laser irradiation of Bil containing VP, G3), Bil (Bil without laser irradiation, G4), and Bil irradiated with laser for 1 (G5), 5 (G6), 10 (G7), 15 (G8), and 20 (G9) minutes, along with VP irradiated with laser for 15 minutes. Sterilized samples were co‑cultured with *E. coli* or *S. aureus* suspensions (1 × 10⁶ CFU/mL) in 24‑well plates. The mixtures were incubated at 37 °C for 24 hours. The bacterial suspensions were serially diluted, plated onto LB agar plates (with three replicates per group), and incubated for an additional 24 hours. Colonies were counted using an automatic colony counter.

Bacterial suspensions (1×10⁷ CFU/mL) were mixed with sterilized samples and irradiated as above. 24 h incubation, live/dead staining was performed using a BacLight™ Bacterial Viability Kit (Thermo Fisher), with SYTO 9 (green, live cells) and propidium iodide (red, dead cells). Fluorescence images were acquired via inverted fluorescence microscopy (Nikon Eclipse Ti2, 40× objective). Viable/dead cell ratios were quantified using Imagej software. The morphology of bacteria after different treatments was also observed via SEM.

The biofilm inhibition capacity of Bil samples was evaluated against Escherichia coli (Gram-negative) and Staphylococcus aureus (Gram-positive) through crystal violet (CV) quantification and biofilm biomass dissolution assays.Bacterial suspensions (1×10⁷ CFU/mL) were incubated with Bil samples in 96-well plates (200 μL/well) and subjected to laser irradiation (690 nm, 25 mW/cm^2^) for 10 or 15 min. After 36 h incubation (37°C, 120 rpm), planktonic cells were removed by triple PBS washes. Adherent biofilms were stained with 0.1% CV (10 min), washed, air-dried, and crystal Violet Staining Solution-stained biofilms were solubilized in 95% ethanol (100 μL/well, 30 min orbital shaking). Absorbance at 570 nm was measured using a microplate reader (BioTek Synergy H1).Concurrently, bacterial biofilms after 36 hours of co-culture were fluorescently stained using a bacterial live/dead staining kit and imaged under a confocal microscope.

**1.15. DPPH free radical scavenging capacity test**

The antioxidant activity of samples was evaluated via DPPH radical scavenging assay. Briefly, 50 mg of each sample was immersed in ethanol (5 mL) and incubated in the dark for 4 h. The supernatant (500 μL) was mixed with 0.1 mM DPPH ethanolic solution (500 μL) and reacted in the dark for 30 min. Aliquots (200 μL/well, triplicate) were transferred to a 96-well plate, and absorbance at 517 nm was measured using a microplate reader (BioTek Synergy H1). DPPH scavenging activity (%) was calculated as:

$DPPH radical scavenging (\%) =\frac{A1-A0}{A1}$×100

where A1 represents the absorbance value of the control and A0 represents the absorbance value of the sample mixed solution with DPPH.

**1.16. ABTS free radical scavenging capacity test**

The ABTS radical scavenging activity of samples was quantified using a modified ABTS assay. ABTS working solution was prepared by mixing 7 mM ABTS with 2.4 mM potassium persulfate (1:1 v/v) and incubating at 4°C for 12 h. Samples were reacted with ABTS solution (1:5 v/v) at 37°C under light-protected conditions for 30 min. Absorbance at 734 nm (A0) was measured in triplicate using a UV-vis spectrophotometer (Shimadzu UV-2600). Control measurements (A_1_) used ddH_2_O instead of samples. Scavenging activity (%) was calculated as:

$ABTS radical scavenging (\%) =\frac{A1-A0}{A1}$×100

**1.17. Intracellular antioxidant assay**

The intracellular antioxidant capacity of the samples was evaluated through CCK-8 viability assays and ROS fluorescence detection. L929 fibroblasts, RAW264.7 macrophages, and HUVECs were seeded in 24-well plates and pre-cultured for 24 h. Cells were exposed to oxidative stress by replacing the culture medium with 500 μM H_2_O_2_-supplemented medium, while test samples were placed in the upper Transwell chamber. After 2 h co-incubation (37°C, dark), cell viability was quantified using a CCK-8 kitby measuring absorbance at 450 nm.

For ROS level analysis, cells were stained with 10 μM DCFH-DA (Beyotime) under identical treatment conditions. Following 20 min incubation (37°C, 5% CO_2_), intracellular ROS-generated fluorescence (ex/em: 488/525 nm) was imaged using an inverted fluorescence microscope (Nikon Eclipse Ti2, 20× objective). alized to untreated controls.

**1.18. Modulation of Intracellular Reactive Oxygen Species by Composite Hydrogel Systems**

L929 cells were incubated for 24 hours with the shell formulation alone, the shell formulation plus laser irradiation (shell+La), the shell formulation plus laser irradiation and the EGCG-free core (shell+La+b-core), or the shell formulation plus laser irradiation and the EGCG-loaded core (shell+La+core). Following the respective treatments, intracellular reactive oxygen species levels were assessed using 10 μM DCFH-DA (Beyotime) as the fluorescent probe. After a 20-minute incubation (37°C, 5% CO₂), fluorescence resulting from intracellular ROS production was visualized with an inverted fluorescence microscope (excitation/emission: 488/525 nm), and the results were compared to those of the untreated control group.

**1.19. qPCR**

Total cellular RNA was isolated using an RNA extraction kit (Vazyme Biotech, Nanjing, China). cDNA synthesis was performed with the PrimeScript™ RT Reagent Kit (Vazyme Biotech) following the manufacturer's protocol. Quantitative polymerase chain reaction (qPCR) amplification was carried out using SYBR Green Master Mix (Yeasen Biotechnology, Shanghai, China) on a QuantStudio 6 Flex system (Thermo Fisher Scientific). The GAPDH gene served as the endogenous control. Relative gene expression levels were calculated via the 2^−ΔΔCt^method.Inflammation-related markers, including M1 polarization genes (iNOS, TNF-α, IL-6) and M2 polarization genes (Arg-1, IL-10, CD206), were analyzed. All qPCR experiments were conducted in three independent biological replicates. Gene-specific primers (Table S1), designed using Primer-BLAST (NCBI) and validated for specificity via melt curve analysis, were synthesized by Sangon Biotech (Shanghai, China).

| **Target** | **Primer sequence** | |
| --- | --- | --- |
| iNOS | Forward (F) | GGCTCCAGCATGTACCCT |
|  | Reverse (R) | GCCCACTGAGTTCGTCCC |
| TNF-α | F | ATGAGCACAGAAAGCATGATCCG |
|  | R | AGAGGCTGAGACATAGGCAC |
| IL-6 | F | GACTGATGCTGGTGACAACC |
|  | R | AGACAGGTCTGTTGGGAGTG |
| Arg1 | F | CTGGCCTTTGTTGATGTCCCT |
|  | R | CCCAGCACCACACTGACT |
| IL-10 | F | CAACATACTGCTAACCGACT |
|  | R | GGCATCACTTCTACCA |
| CD206 | F | CAAAGCCATGCTGTAGTACCG |
|  | R | CCTTTTCATTTGTGCATGTGT |
| GAPDH | F | CAGTGGCAAAGTGGAGATTGTTG |
|  | R | TCGCTCCTGGAAGATGGTGAT |

Table S1: Primer sequence

**1.20. Diabetic infected wound healing model**

A diabetic wound model was established using 5–6-week-old male C57BL/6J mice (20 ± 2 g, Beijing SPF Biotechnology Co., Ltd.) through intraperitoneal injection of streptozotocin (STZ, 50 mg/kg in 0.1 M citrate buffer, pH 4.5) for five consecutive days after 12 h fasting. Diabetes was confirmed when random blood glucose levels from tail vein sampling exceeded 16.7 mmol/L. All procedures were approved by the Animal Ethics Committee of the Second Affiliated Hospital of Kunming Medical University (Approval No. KYFEYXM2024165). Full-thickness dorsal wounds (6 mm diameter) were surgically created using ophthalmic scissors. Mice were divided into five groups: (1) Control (10 μL PBS), (2) Core (SF-Gel nanofibers loaded with EGCG), (3) Shell (PVA-TSPBA hydrogel encapsulating 30 μg/mL VP and 100 μg/mL exosomes +laser irradiation), (4) Bil (bilayer hydrogel combining SF-Gel nanofibers loaded with EGCG and PVA-TSPBA hydrogel encapsulating 30 μg/mL VP and 100 μg/mL exosomes without irradiation), and (5) Bil+La (bilayer hydrogel combining SF-Gel nanofibers loaded with EGCG and PVA-TSPBA hydrogel encapsulating 30 μg/mL VP and 100 μg/mL exosomes with laser irradiation).Twenty-four hours prior to treatment,each wound was inoculated with a 10 μL mixed bacterial suspension containing *Staphylococcus aureus* and *Escherichia coli* at a concentration of 1 × 10⁸ CFU/mL.All wounds were covered with Tegaderm™ films (3M Healthcare) to prevent wound contraction during healing.Wound closure was monitored at days 0, 3, 7, and 14 post-treatment via digital photography (Canon EOS 90D) and quantified using ImageJ software (v1.53). Harvested wound tissues were fixed in 4% paraformaldehyde, paraffin-embedded, sectioned (5 μm), and subjected to H&E, Masson’s trichrome, Picrosirius red staining, and immunofluorescence for Ki67 (proliferation), CD86/CD163 (macrophage polarization), CD31 and α-SMA(angiogenesis). Digitized slides (Pannoramic MIDI scanner, 3DHISTECH) were analyzed using ImageJ to quantify histological parameters. Data are presented as mean ± SD from three independent experiments.

**1.21. Transcriptome Sequencing**

For RNA-seq analysis, mice wound samples from the control and Bil+La groups were collected at day 14 post-surgery, and each group comprised at least three samples. Total RNA was extracted from the tissue samples using TRIzol® Reagent according the manufacturer’s instructions (Vazyme) and genomic DNA was removed using DNase I. The next RNA sequencing was conducted using the Illumina NovaSeq X Plus platform (Shanghai Biotechnology Co., Ltd, China). Differential expression (DESeq2: |log₂FC|>1, pvaule<0.05) and functional enrichment (KEGG/GSEA; |NES|>1, p<0.05) were analyzed. Heatmaps visualized clustered DEGs (pheatmap).

**1.22. Rabbit Ear Scar Model Experiment**

To evaluate the anti-scarring efficacy of the samples, we established a reproducible and quantifiable hypertrophic scar model in New Zealand white male rabbits (3-4 months old) following previously reported methods [6]. The rabbits were anesthetized via marginal ear vein injection of pentobarbital sodium (30 mg/kg). Full-thickness wounds (7 mm diameter) were created on the ventral side of each ear by surgically removing the epidermis, dermis, and perichondrium using fine forceps and ophthalmic scissors to ensure scar hyperplasia during healing. The wounds were treated with: (1) Control (10 μL PBS), (2) VP (10 μL, 30 mg/mL verteporfin), (3) b-Bil (VP-free bilayer hydrogel), or (4) Bil (VP-loaded bilayer hydrogel laser irradiation). All wounds were covered with Tegaderm™ film until complete re-epithelialization.

Wound healing progression was documented via digital photography at days 0, 5, 10, and 30 post-treatment. On day 30, scar tissues were harvested, fixed in 4% paraformaldehyde, paraffin-embedded, and sectioned for histological analysis, including H&E staining (tissue morphology), Masson’s trichrome (collagen deposition), and Picrosirius red staining (collagen typing under polarized light). Digital slide scanning (Pannoramic MIDI, 3DHISTECH) and ImageJ (v1.53) were used to quantify collagen content (%) and the type I/III collagen ratio. Scar elevation index (SEI) was calculated for morphometric analysis. All procedures were approved by the Animal Ethics Committee of Kunming Medical University (Approval No. KMMU20241906).

**1.23. Statistical Analysis**

Data were represented as mean ± SD values from a minimum of three independent experiments. Statistical analysis was performed via GraphPad Prism software (version 9.5.0) by using one-way analysis of variance (ANOVA) with Tukey comparison for multiple groups. Statistical significance with varying levels was defined as follows: *p<0.05, **p < 0.01, ***p < 0.001, and ****p < 0.0001.

1. **Supplementary Figures**

| Abbreviation | Full name |
| --- | --- |
| VP | Verteporfin |
| EGCG | Epigallocatechin gallate |
| SF | Silk Fibroin |
| Gel | Gelatin |
| Core | EGCG-loaded silk fibroin/gelatin nanofibers |
| b-Core | silk fibroin/gelatin nanofibers |
| shell | PVA‑TSPBA hydrogel loaded with VP and Exos@PLGA |
| b-Bil | Double-layer composite hydrogel without drug loading |
| Bil | Drug-loaded double-layer composite hydrogel |

Table S2: Abbreviations for different structures and components


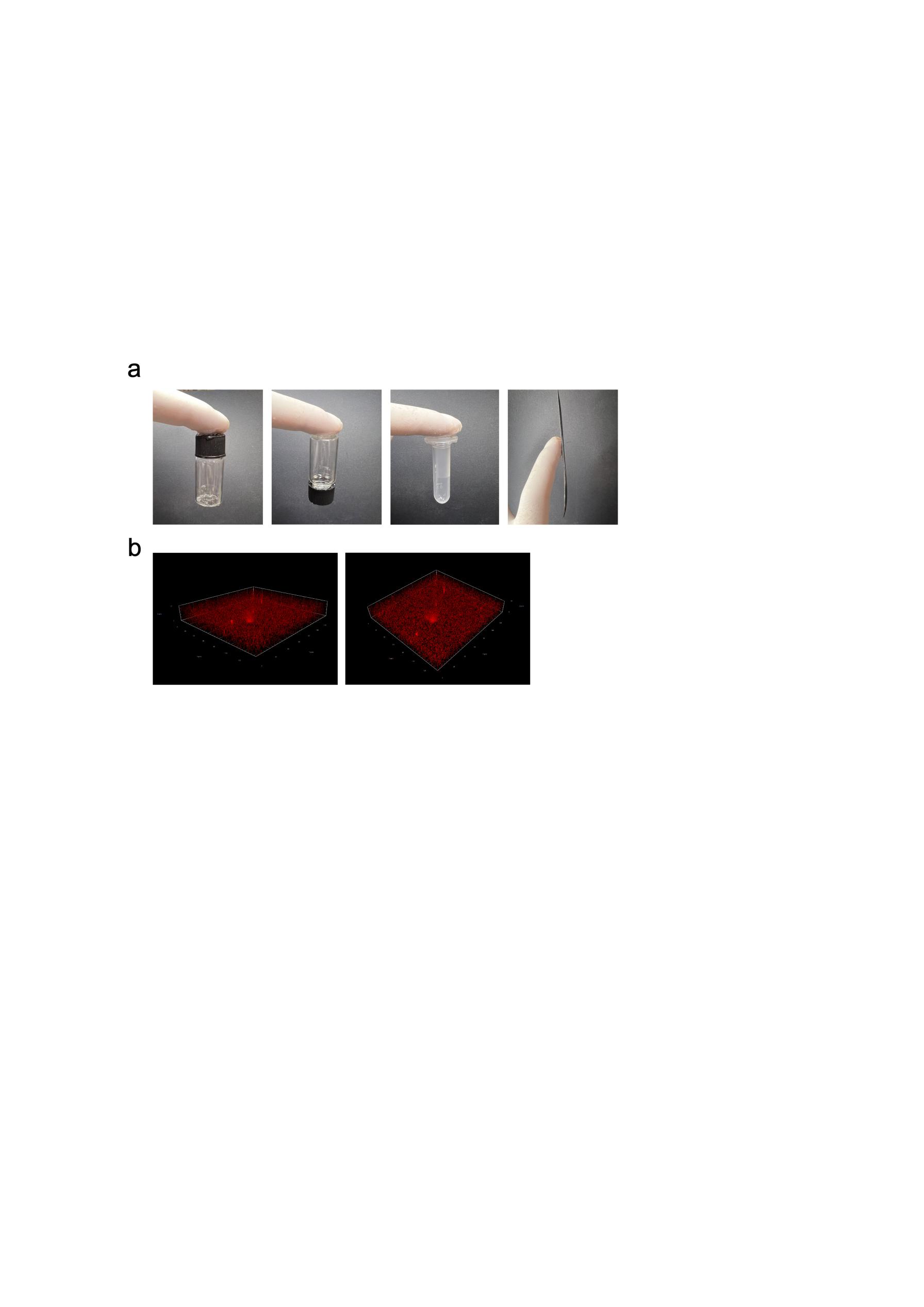


**Figure S1.**Adhesive Properties of PVA-TSPB. (a) Photographs demonstrating the adhesion of PVA-TSPB hydrogel to plastic, glass, and metal substrates. (b) Three-Dimensional Distribution of PLGA-Encapsulated Exosomes within PVA-TSPBA Hydrogel (shell).


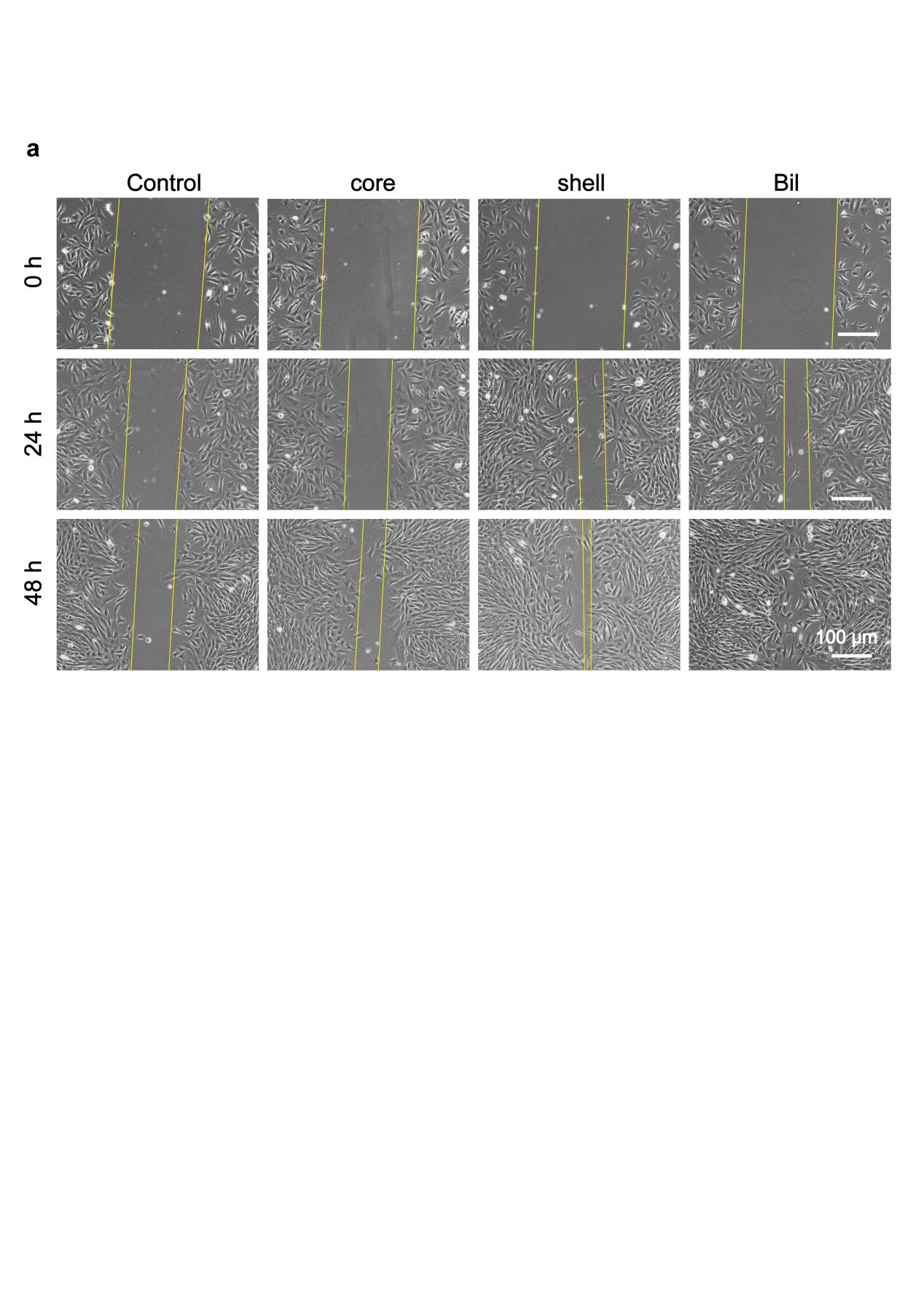


**Figure S2.** effects on Vascular Endothelial Cells. (a) Representative images showing Vascular Endothelial Cells migration under different treatment conditions.


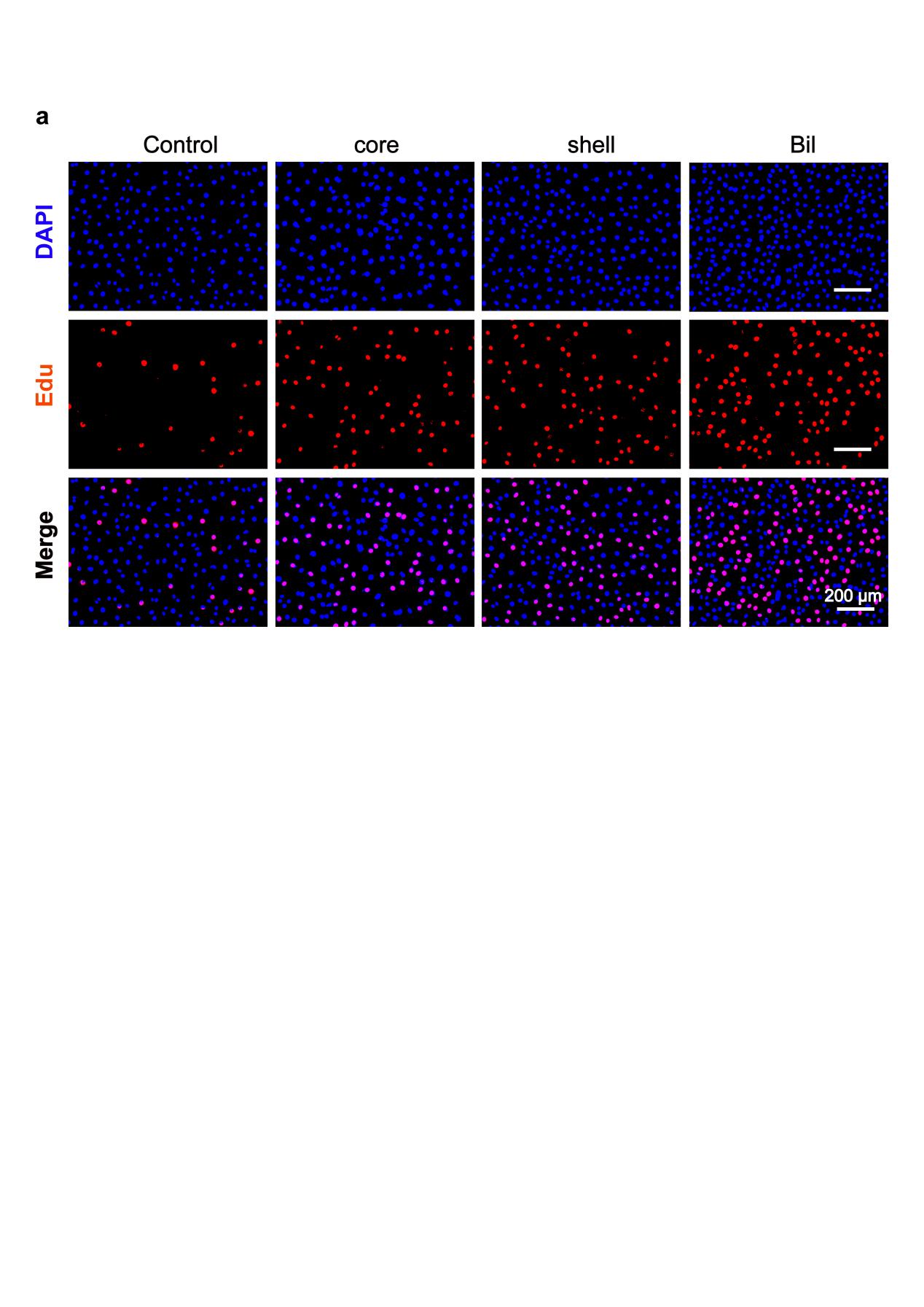


**Figure S3.** effects on Vascular Endothelial Cells. (a) Evaluation of Vascular Endothelial Cells proliferation using EdU staining across experimental groups.


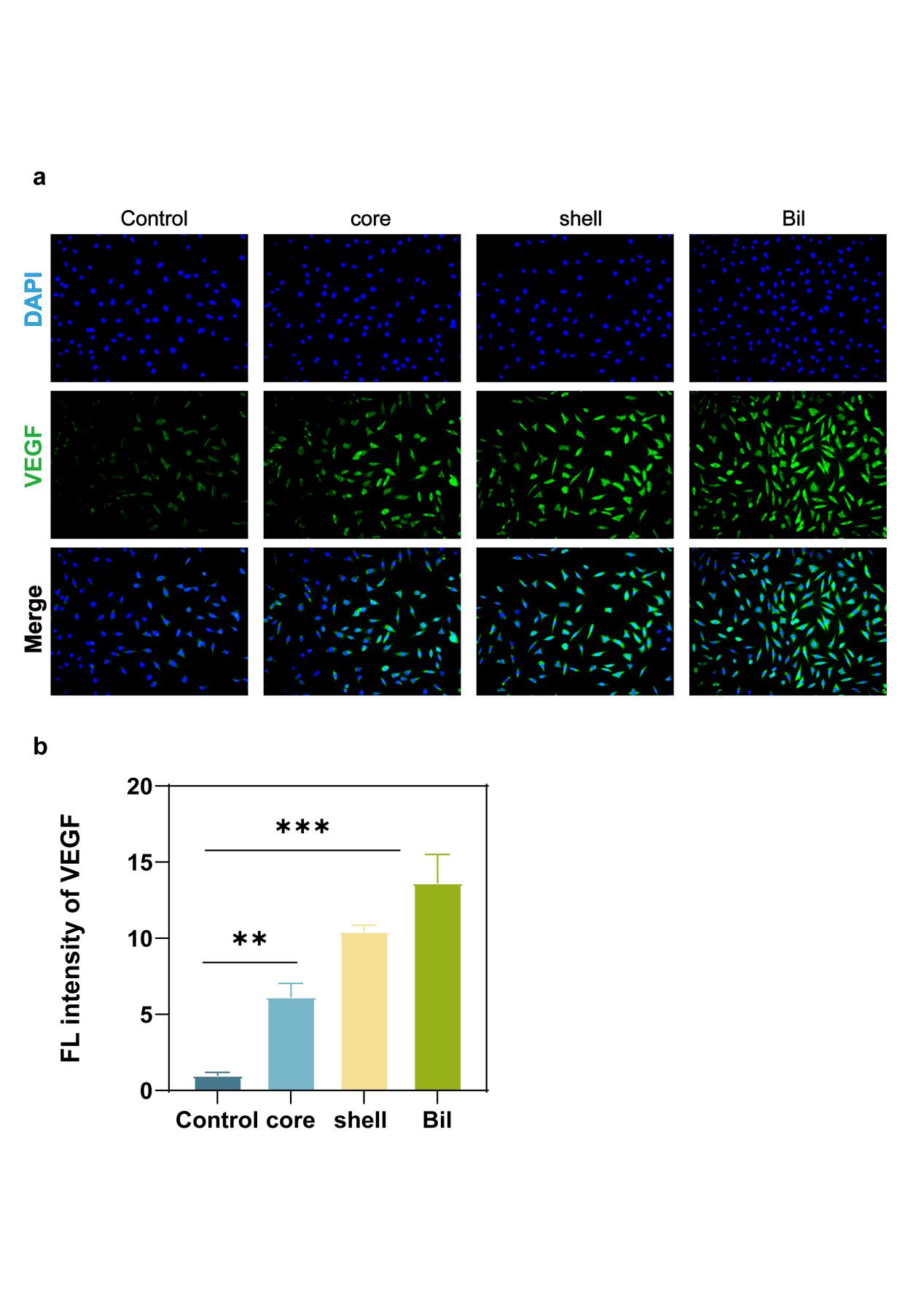
**Figure S4.** effects on Vascular Endothelial Cells. (a) Quantification of VEGF secretion from vascular endothelial cells based on fluorescence detection. (b) Comparative analysis of data under different treatment conditions.


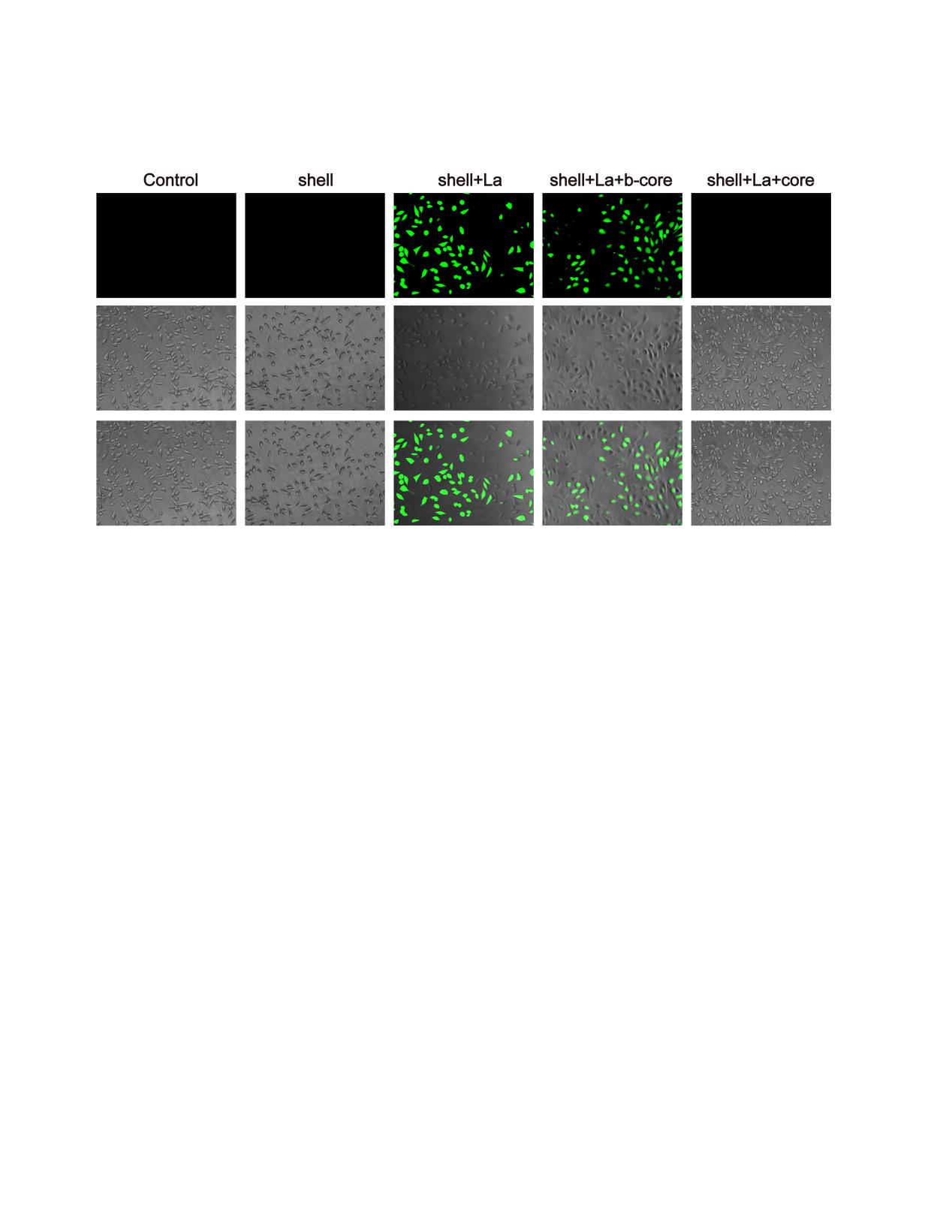
**Figure S5.** Intracellular oxidative stress in different treatment groups was assessed using the DCFH-DA fluorescent probe.(a) Oxidative stress levels in control, shell, shell+La, shell+La+b-core, and shell+La+core groups were evaluated by DCFH-DA staining.


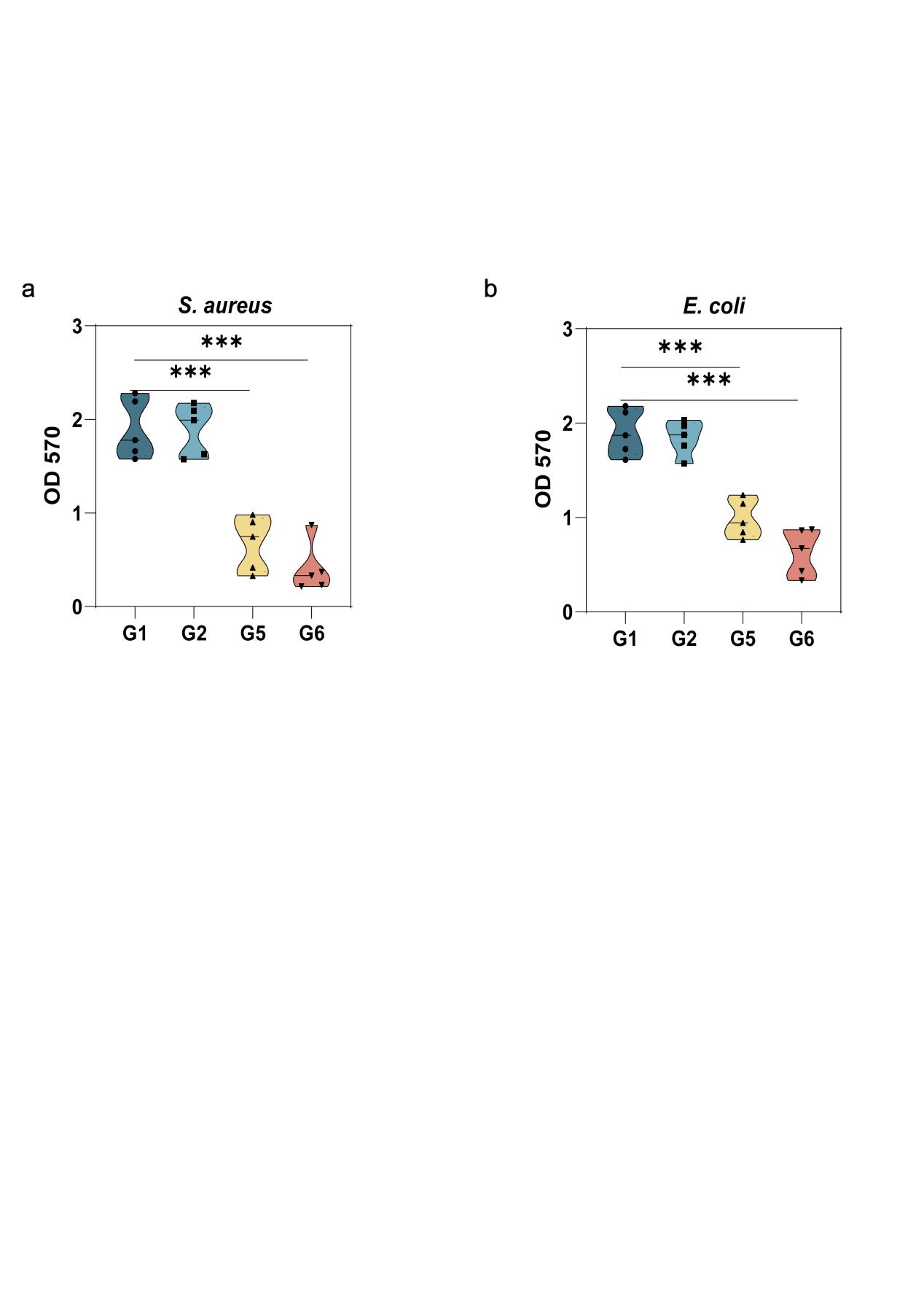


**Figure S6.** Antibacterial Function of the Bil Composite Hydrogel. (a) Quantification of biofilm biomass (OD 570 nm) for *S. aureus* after crystal violet staining. (b) Quantification of biofilm biomass (OD 570nm) for *E. coli* after crystal violet staining.


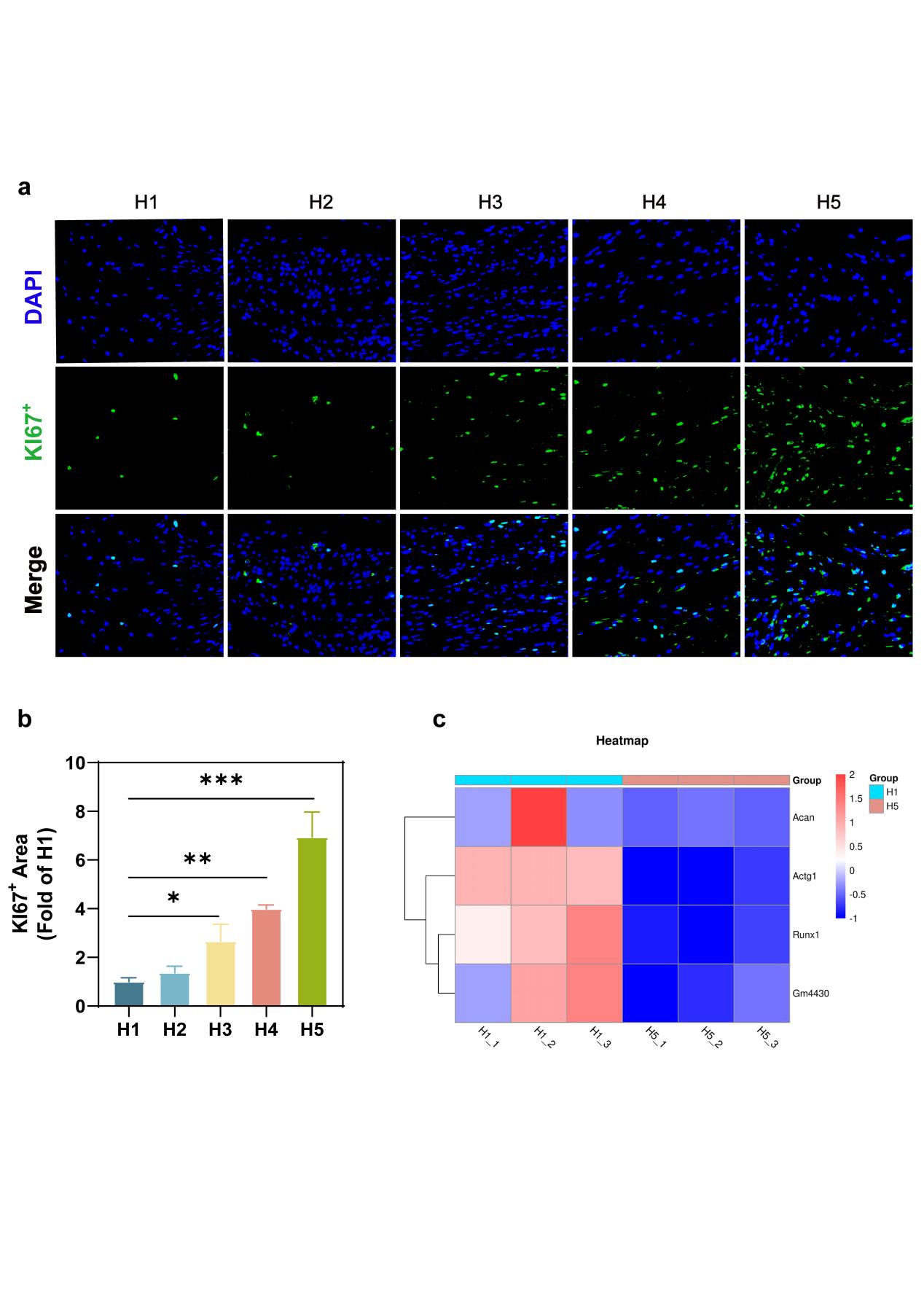
**Figure S7.** Evaluation of the Mechanisms of Bil in Promoting Diabetic Wound Healing. (a) Immunofluorescence staining of proliferating cells (KI67^+^)in the wound tissue at day 14. (b) Quantitative analysis of KI67^+^ expression levels in wound areas. (c) Significantly differentially expressed genes linked to fibrosis-related genes after Bil+La treatment.

1. Li, P. *et al.* “Exosomes derived from umbilical cord mesenchymal stem cells protect cartilage and regulate the polarization of macrophages in osteoarthritis.” Ann Transl Med 10, no. 18 (2022): 976. <https://doi.org/10.21037/atm-22-3912>
2. Wang, R., Liu, L., Han, F., Ma, Q. & He, H. “Exosomes derived from human umbilical cord mesenchymal stem cells can reverse ventricular remodeling and improve long-term cardiac function after acute myocardial infarction.” Biochemical and Biophysical Research Communications 768, no. (2025): 151920. <https://doi.org/10.1016/j.bbrc.2025.151920>
3. Xie, B. *et al.* “Progesterone PLGA/mPEG-PLGA Hybrid Nanoparticle Sustained-Release System by Intramuscular Injection.” Pharmaceutical Research 35, no. 3 (2018): 62. <https://doi.org/10.1007/s11095-018-2357-x>
4. Yang, J. *et al.* “Copper ion/gallic acid MOFs-laden adhesive pomelo peel sponge effectively treats biofilm-infected skin wounds and improves healing quality.” Bioact Mater 32, no. (2024): 260-276. <https://doi.org/10.1016/j.bioactmat.2023.10.005>
5. Rockwood, D. N. *et al.* “Materials fabrication from Bombyx mori silk fibroin.” Nature Protocols 6, no. 10 (2011): 1612-1631. <https://doi.org/10.1038/nprot.2011.379>
6. Cao, L. L. *et al.* “A preliminary study on ultrasound techniques applied to evaluate the curative effect of botulinum toxin type a in hypertrophic scars.” Heliyon 10, no. 15 (2024): e34723. <https://doi.org/10.1016/j.heliyon.2024.e34723>
